# Partnered health research in Canada: a cross-sectional survey of perceptions among researchers and knowledge users involved in funded projects between 2011 and 2019

**DOI:** 10.1186/s12961-025-01299-8

**Published:** 2025-03-03

**Authors:** Kathryn M. Sibley, Leah K. Crockett, Heather L. Gainforth, Ian D. Graham, Femke Hoekstra, Jeff S. Healey, Masood Khan, Sara Kreindler, Kent C. Loftsgard, Christopher B. McBride, Kelly J. Mrklas, Alexie J. Touchette

**Affiliations:** 1 Department of Community Health Sciences, University of Manitoba, 379-753 McDermot Avenue, Winnipeg, MB R3E 0W3 Canada; 2 George & Fay Yee Centre for Healthcare Innovation, 379-753 McDermot Avenue, Winnipeg, MB R3E 0W3 Canada; 3 School of Health and Exercise Sciences, University of British Columbia, ART360-1147 Research Road, Kelowna, BC V1V 1V7 Canada; 4 International Collaboration On Repair Discoveries, University of British Columbia, ART360-1147 Research Road, Kelowna, BC V1V 1V7 Canada; 5 School of Epidemiology and Public Health and School of Nursing, University of Ottawa, 501 Smyth Road, Ottawa, ON K1G 5Z3 Canada; 6 Centre for Implementation Research, Ottawa Hospital Research Institute, 501 Smyth Road, Ottawa, ON K1G 5Z3 Canada; 7 Department of Medicine, Division of Social Medicine, University of British Columbia, 1088 Discovery Avenue, Kelowna, BC V1V 1V7 Canada; 8 Centre for Chronic Disease Prevention and Management, Southern Medical Program, University of British Columbia, 1088 Discovery Avenue, Kelowna, BC V1V 1V7 Canada; 9 Population Health Research Institute, McMaster University, 1280 Main Street West, Hamilton, ON L8S 4L8 Canada; 10 CIHR Strategy for Patient Oriented Research, #106-105 2nd St. West, North Vancouver, BC V7M 0E3 Canada; 11 Spinal Cord Injury British Columbia, 780 SW Marine Drive, Vancouver, BC V6P 5Y7 Canada; 12 Alberta Health Services, 3D10, 3280 Hospital Drive NW, Calgary, AB T2N 4Z6 Canada

**Keywords:** Research partnerships, Integrated knowledge translation, Community-based participatory research, Action research, Patient-oriented research

## Abstract

**Background:**

Engaging knowledge users in health research is accelerating in Canada. Our objective was to examine perceptions of partnered health research among individuals involved in funded Canadian partnered health research projects between 2011 and 2019.

**Methods:**

We invited 2155 recipients of 1153 funded projects to answer a questionnaire probing project characteristics and perceptions of partnered health research. We described and compared perceived effects of involving knowledge users in the project, team cohesion, capability, opportunity and motivation for working in partnership between two categories of respondents: project role [nominated principal investigators (NPIs), other researchers and knowledge users] and gender.

**Findings:**

We analysed data from 589 respondents (42% NPIs, 40% other researchers and 18% knowledge users; 56% women). Among the perceived effects variables, the proportion of ratings of significant influence of involving knowledge users in the project ranged between 12% and 63%. Cohesion, capability, opportunity and motivation variables ranged between 58% and 97% agreement. There were no significant differences between respondent groups for most variables. NPIs and women rated the overall influence of involving knowledge users as significant more than other respondent groups (*p* < 0.001). NPIs also reported higher agreement with feeling sufficiently included in team activities, pressure to engage and partnerships enabling personal goals (all *p* < 0.001).

**Conclusions:**

Most respondents held positive perceptions of working in partnership, although ratings of perceived effects indicated limited effects of involving knowledge users in specific research components and on project outcomes. Continued analysis of project outcomes may identify specific contexts and partnership characteristics associated with greater impact.

**Supplementary Information:**

The online version contains supplementary material available at 10.1186/s12961-025-01299-8.

## Introduction

Partnerships between health researchers and knowledge users, which involve individuals and organizations from interested and/or affected groups in the research process, is recommended to increase the relevance of research findings, impact in society, and potential uptake in healthcare practices and decision-making [1, 2]. A host of approaches, including but not limited to integrated knowledge translation (IKT) [3], patient-oriented research (POR) [4] and community-based participatory research (CBPR) [5], can guide partnered health research and are often supported and encouraged by health research funding agencies [6]. However, data on partnered health research reflects a patchwork of evidence. For example, Hoekstra et al.’s 2020 review of reviews on health research partnerships was able to extract data on the nature of stakeholder engagement in just 21% of reviews and identified the lack of reporting on partnership processes as a key issue [7, 8]. Mrklas et al.’s 2022 systematic review of health research partnership outcomes and impacts found that only 38% of studies reported outcomes and impacts, of which most were positive, but few were explicitly defined and often combined constructs [9, 10]. Reviews of published partnered health research are important but are limited by reporting practices. Calls are growing for a comprehensive and deeper understanding of collaborative partnership processes and their intermediate and long-term outcomes to optimize the conduct and impact of partnered health research [11].

The last large study of partnered health research in Canada was undertaken in 2011. The Canadian Institutes of Health Research (CIHR), a national funding agency, undertook an evaluation of targeted funding opportunities between 2005 and 2010 that required knowledge user involvement on grant proposals [12]. This analysis compared researcher and knowledge user attitudes and perceptions of partnering, identified beneficial effects of involving research users on specific components of the research process, and identified positive beliefs about the potential impact of partnered research. This analysis also identified a gender difference in which more women held partnered research grants compared with men (a reverse pattern from the general pool of CIHR grants at that time) [13]. Since this evaluation was conducted, theoretical and conceptual foundations for partnering have evolved [14], as have funding supports, expectations [15] and calls for increased consideration of gender in knowledge translation [16], thus presenting the need and opportunity for an updated comprehensive assessment of Canadian partnered health research.

Our overarching aim is to improve the understanding of partnered health research to inform partnership practice recommendations for undertaking partnered research. Our preliminary work identified 1153 federally and provincially funded Canadian health research projects that included a partnership between 2011 and 2019 [17]. Using publicly available data, we determined that projects addressed many fields of health. The types of research primarily focused on health and social care services, receiving funding for longer periods and larger amounts between 2011–2013 and 2017–2019. Analysis of funded health research is important as competitive funding via peer review is one indicator of research quality. However, publicly available data in Canada are restricted to basic information, such as project titles and abstracts, funding amounts and durations. Our identification of 1153 Canadian partnered health research projects offers an important opportunity to learn more about how each project was undertaken and the perceived effects of doing so. Given the known limitations of reporting in partnered research [7, 8], relying on published articles is insufficient. Primary data collection can contribute to filling these important evidence gaps. In particular, examining differences between partnership team roles (that is, researchers and knowledge users) can provide insight into trends over time, and exploring gender differences in perceptions is appropriate in light of the gendered distribution of partnered health research.

We endeavoured to expand the understanding of partnered health research through a survey-based study of self-reported practices, perceived effects and perceptions of involving knowledge users in the 1153 funded Canadian partnered health research projects funded between 2011 and 2019. In this manuscript, we describe survey administration procedures and respondent characteristics and compare perceived effects, team cohesion, capability, opportunity and motivation for working in partnership between two categories of respondents: project role and gender.

## Methodology and methods

### Context and conceptual foundations

This study is a component of a set of research projects to understand current practices in Canadian partnered health research and develop recommendations for undertaking partnered research (CIHR grant PJT #156372). Sampling and data collection took place in 2020. We took a pragmatic approach [18], focusing on the the potential utility of our findings to address real-world problems [19]. We used IKT as our partnership approach. Our team includes people with lived experience of a health condition, people with lived professional experience (including health professionals and knowledge translation practitioners), community organization and research funding organization representatives, trainees and academic researchers who practice and/ or study health research collaborations with knowledge users. All team members were invited to contribute to the projects to the extent that they would like to be involved and were engaged regularly through email updates and requests for input and meetings (minimum of once per year, but often more frequently). Involvement of team members on project components varied between and within individuals, typically spanning levels consistent with Consult, Involve and/or Collaborate on the IAP2 Spectrum of Public Participation [20]. We obtained research ethics approval from the University of Manitoba Health Research Ethics board and followed published recommendations for survey reporting [21].

### Study design

We used a cross-sectional design consisting of an online self-report questionnaire.

### Participants and sampling

Individuals named as recipients of the 1153 Canadian federally and provincially funded partnered health research projects between 2011 and 2019 were eligible to participate. We acquired project data (project name, year funded and funding value) and names of recipients from publicly available data where possible [22] or from funding agencies. We included individuals with principal investigator (PI) status at the time of submission. PIs were defined as individuals who lead the intellectual direction of the research project. An individual holding a PI role may be an academic researcher or knowledge user. Consistent with Canadian funding reporting practices, we considered the first PI named as the nominated principal investigator (NPI), defined as the individual responsible for coordinating the financial and administrative aspects of the research project as well as leading the intellectual direction of the proposed activities [23]. In projects where only one PI was named (an NPI), we also included individuals with co-investigator status. Individuals associated with more than one project were included only for the project on which they held an NPI role or the project with the earliest year of funding (and highest likelihood of project completion).

### Recruitment strategy

We searched for publicly available email addresses for eligible individuals, then used a recruitment approach guided by Dillman [24]. Eligible individuals received an introductory message from K.M.S. with a pre-notice informing them about the study. The invitation indicated the recipient had been identified as having a role on an eligible project. The second message provided a unique link to the questionnaire. Two reminders were sent to nonresponders as needed. The questionnaire and all communications were available in English and French, as the two official languages of Canada.

### Questionnaire instrument

We developed a customized questionnaire because no standardized instruments existed that examined our constructs of interest. Questionnaire content (including domains of interest, specific questions and response options) were informed by published definitions, concept papers, descriptions of partnered research practices and processes and related instruments, where available [25–33]. Questionnaire design decisions were informed by response scale evidence where possible [34]. K.M.S. drafted the questionnaire, which underwent multiple rounds of review for content validation by project team members and revision until consensus on content and wording was achieved. We conducted pilot testing with 12 individuals purposively selected to maximize representation related to our sample population. Individuals completed the questionnaire independently, then provided feedback on questionnaire clarity, content and format. We reviewed feedback after each pilot and revised the questionnaire until no further actionable feedback was reported.

The final questionnaire (Appendix 1) included nine sections: (1) introduction and consent; (2) eligibility; (3) role and project details; (4) respondent characteristics; (5) partnership practices; (6) perceived effects of involving knowledge users in the project; (7) team cohesion; (8) capability, opportunity and motivation for working in partnership and (9) knowledge user experiences. The eligibility section included data to orient respondents to the eligible project, and respondents were asked to confirm if the project involved knowledge users throughout the research process. Respondents were instructed to answer in relation to the eligible project. We used branching logic to customize questions by project role (researcher or knowledge user), project status (completed or ongoing), research components included in project, knowledge user involvement in included components of the research process (binary yes/ no), and perceived effects of knowledge user involvement (completed projects or project components only). The questionnaire’s evaluative dimensions included item-specific variables for sections assessing respondent and project characteristics and practices and Likert-type questions for sections addressing perceived effects (unipolar 4-point scale with descriptive anchors: significant influence, moderate influence, a little influence, no influence). In addition, it included ratings of team cohesion and capability, opportunity and motivation for working in partnership (bipolar 5-point scale with descriptive anchors: strongly agree, agree, neutral, disagree and strongly disagree). Categorical variables with closed-ended responses included an “I don’t know” option and options for additional responses. Questionnaire variables, response options and branching logic are detailed in Appendix 2. The questionnaire took approximately 20 min to complete.

### Data processing and analysis

Data analysis was conducted with SPSS version 28.0. We coded all responses for level of completion and conducted sensitivity analyses comparing (i) respondents and nonrespondents and (ii) respondents with partial versus complete data, using chi-squared or independent *t*-tests. We used publicly available data (funder, year of funding and amount of funding) as well as respondent role and project status for respondents with partial versus complete data.

We included all respondents who answered up to the question which we determined to represent the minimum amount of analysable data in the final analysis (question 4.3: knowledge user involvement in included components of the research process). We made this decision to retain as much meaningful data as possible. We reviewed open-ended responses and recoded and collapsed categories as appropriate. We merged variables separated from branching logic as appropriate.

We calculated summary statistics for respondent characteristics (four items: gender identity, member of visible minority in Canada, Indigenous and project role); perceived effects of involving research users in project (five items: overall influence on the project, influence on project outcomes – production of useful research findings, promotion of evidence-informed decision-making in healthcare, project impact on health care professional practices and project impact on health system policies); perceived effects of involving research users in specific project components (eight items: setting research priorities, choosing research questions, developing study design and methods, choosing study outcomes, development of research ethics documents, participant recruitment, data collection, data analysis and interpretation, dissemination of findings to academic audiences and dissemination of findings to non-academic audiences) and team cohesion (nine items from [35]); capability, opportunity and motivation for working in partnership (10 items) [36].

We compared the proportion of respondents rating perceived effects variables as having a significant influence with chi-squared tests, using predicted proportions for the expected values between respondent project role (NPIs, other researchers and knowledge users) and gender idenitity (women and men). We were unable to compare other gender identities owing to low cell counts. To account for instances of low cell counts, we collapsed Likert ratings for team cohesion, capability, opportunity and motivation for working in partnership variables into three categories: agree/neutral/disagree, and used chi-squared tests to compare differences in proportion of respondent ratings for respondent project role and gender. Consistent with recommendations for considering risk of type 1 error with multiple statistical tests [37], we set a statistical significance level of *p* < 0.001.

## Results

### Recruitment (Fig. 1)

**Fig. 1:**
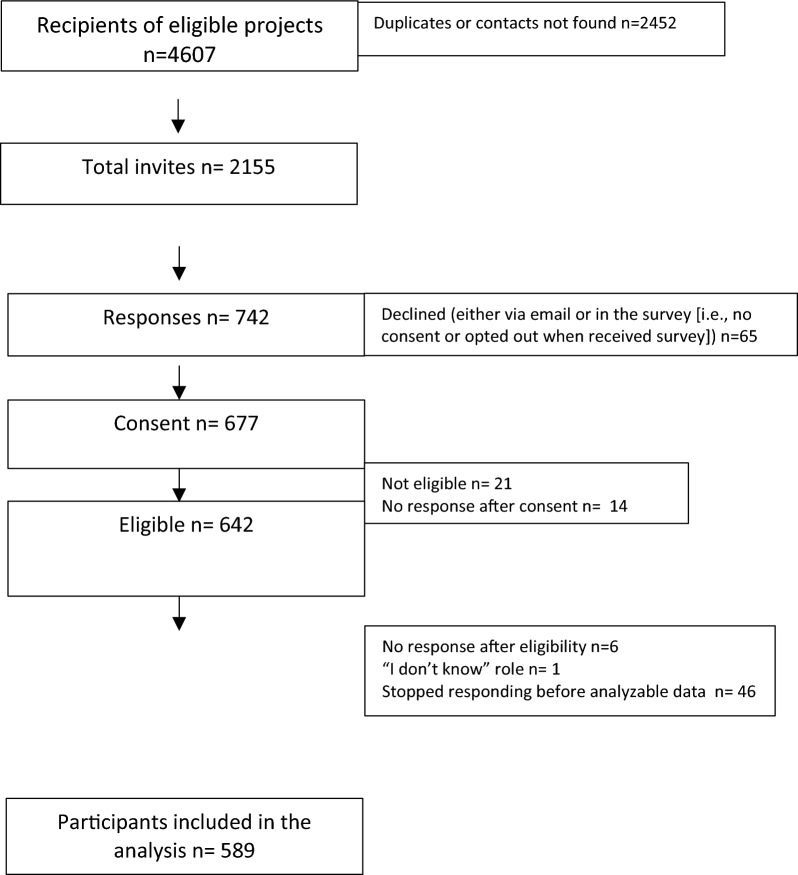
Recruitment flow

Data collection occurred between August and October 2020. We invited 2155 individuals to participate. We received a reply from 742 individuals (34% of recipients). We removed those who declined (*n* = 65, 9% of those who replied), indicated they were ineligible (*n* = 21, 1%) and those who stopped responding before any analysable data was provided (*n* = 67, 9%). Data from 589 respondents were included (92% of eligible respondents). Sensitivity analyses indicated no significant differences in funder, funding year, or amount of funding between respondents and nonrespondents (all *p* > 0.05) and respondents with complete (*n* = 535) versus partial (*n* = 54) data (all *p* > 0.05). There was also no difference in respondent role or project status for respondents with partial versus complete data (both *p* > 0.05).

### Respondent characteristics (Table 1)

**Table 1:** Respondent characteristics (*n* = 589)

		*N* (%)
Gender identity	Woman	328 (56%)
	Man	170 (29%)
	Fluid	1 (0.1%)
	Prefer not to answer	31 (5%)
	Missing	59 (10%)
Member of a visible minority in Canada	Yes	59 (10%)
	No	452 (77%)
	Prefer not to answer	23 (4%)
	Missing	55 (9%)
Indigenous	Yes	23 (4%)
	No	496 (84%)
	Prefer not to answer	16 (3%)
	Missing	53 (9%)
Project role	Nominated principal investigator researcher	248 (52%)
	Other researcher	233 (48%)
	Knowledge user	108 (18%)
	Health system decision or policy maker	23 (21%)
	Healthcare manager or administrator	20 (19%)
	Community organization representative	20 (19%)
	Health professional	14 (13%)
	Technical or research support professional	11 (10%)
	Health professional organization representative	6 (6%)
	Person with lived experience of health condition	5 (5%)
	Knowledge translation professional	5 (5%)
	Other	3 (3%)
	Missing	1 (0.1%)

The majority of respondents identified their gender as woman (*n* = 328, 56%). A small proportion of respondents identified as a member of a visible minority in Canada (*n* = 59, 10%), and 4% (*n* = 23) identified as Indigenous. The majority of respondents identified as researchers who held the role of NPI on the eligible project (*n* = 248, 52%). Many roles were represented among the 108 respondents (18%) who identified as knowledge users on the eligible project: most frequently, health system decision or policy makers (21%), healthcare managers or administrators (19%) or community organization representatives (19%). 456 projects were represented among the 589 respondents. Of these, 365 projects were represented by a single respondent, and 91 projects were represented by multiple respondents (*n* = 224 respondents, range 2–6 respondents per project).

### Perceived effects of involving knowledge users in partnered health research (Tables 2 and 3)

**Table 2:** Proportion of respondents rating perceived influence of knowledge user involvement on outcome as significant, by respondent project role

Outcome	*N* (%) of respondents who perceived a significant influence of knowledge user involvement on outcome				Chi-squared, *p*-value
	Full sample	Project role			
		NPI	Researcher	Knowledge user	
Overall influence (*n* = 548)	313 (57.1)	155 (66.2)	107 (50.2)	51 (50.5)	***χ***^2^** = 13.88**, *p* < 0.001
Setting research project’s priorities (*n* = 428)	208 (48.6)	103 (53.9)	73 (46.2)	32 (40.5)	*χ*^2^ = 4.61, *p* = 0.10
Choosing the research questions (*n* = 386)	170 (44.0)	79 (44.1)	66 (47.8)	25 (36.2)	*χ*^2^ = 2.51, *p* = 0.29
Developing study design and methods (*n* = 349)	118 (33.8)	66 (39.1)	31 (26.7)	21 (32.8)	*χ*^2^ = 4.71, *p* = 0.10
Choosing study outcomes (*n* = 360)	140 (38.9)	75 (44.6)	40 (31.3)	25 (39.1)	*χ*^2^ = 5.48, *p* = 0.06
Developing research ethics documents (*n* = 179)	63 (35.2)	32 (39.0)	20 (30.8)	11 (34.4)	*χ*^2^ = 1.10, *p* = 0.58
Participant recruitment (*n* = 290)	183 (63.1)	92 (65.7)	62 (62.6)	29 (56.9)	*χ*^2^ = 1.27, *p* = 0.53
Data collection (*n* = 241)	123 (51.0)	63 (55.3)	42 (50.6)	18 (40.9)	*χ*^2^ = 2.63, *p* = 0.27
Data analysis and interpretation (*n* = 289)	102 (35.3)	59 (41.0)	25 (25.5)	18 (38.3)	*χ*^2^ = 6.33, *p* = 0.04
Disseminating findings to non-academic audiences (*n* = 342)	171 (50.0)	86 (50.6)	51 (46.4)	34 (54.8)	*χ*^2^ = 1.19, *p* = 0.55
Disseminating findings to academic audiences (*n* = 274)	89 (32.5)	42 (30.0)	27 (31.0)	20 (42.6)	*χ*^2^ = 2.65, *p* = 0.27
The production of useful findings in the field (*n* = 267)	125 (46.8)	58 (50.9)	41 (40.2)	26 (51.0)	*χ*^2^ = 2.91, *p* = 0.23
The promotion of evidence-informed decision-making in health care or the healthcare system (*n* = 267)	81 (30.3)	39 (33.9)	23 (23.0)	19 (36.5)	*χ*^2^ = 4.19, *p* = 0.12
The project’s impact on health care professional practices (*n* = 264)	61 (23.1)	28 (24.8)	19 (19.2)	14 (26.9)	*χ*^2^ = 1.46, *p* = 0.48
The project’s impact on health system policies (*n* = 266)	33 (12.4)	17 (14.9)	8 (8.0)	8 (15.4)	*χ*^2^ = 2.87, *p* = 0.24

Significant values are shown in bold

**Table 3:** Proportion of respondents rating perceived influence of knowledge user involvement on outcome as significant, by gender

Outcome	*N* (%) of respondents who perceived a significant influence of knowledge user involvement on outcome		Chi-squared, *p*-value
	Woman	Man	
Overall influence (*n* = 494)	212 (65.0)	77 (45.8)	***χ***^2^** = 16.80**, *p* < 0.001
Setting research project’s priorities (*n* = 390)	140 (52.2)	49 (40.2)	*χ*^2^ = 4.89, *p* = 0.03
Choosing the research questions (*n* = 349)	113 (47.7)	39 (34.8)	*χ*^2^ = 5.12, *p* = 0.02
Developing study design and methods (*n* = 320)	75 (34.6)	30 (29.1)	*χ*^2^ = 0.96, *p* = 0.33
Choosing study outcomes (*n* = 331)	97 (42.0)	31 (31.0)	*χ*^2^ = 3.56, *p* = 0.06
Developing research ethics documents (*n* = 163)	41 (39.8)	16 (26.7)	*χ*^2^ = 2.88, *p* = 0.09
Participant recruitment (*n* = 262)	117 (65.0)	47 (57.3)	*χ*^2^ = 1.42, *p* = 0.23
Data collection (*n* = 212)	82 (56.9)	27 (39.7)	*χ*^2^ = 5.50, *p* = 0.02
Data analysis and interpretation (*n* = 265)	78 (43.1)	16 (19.0)	***χ***^**2**^** = 14.49**, ***p*** < **0.001**
Disseminating findings to non-academic audiences (*n* = 310)	115 (55.0)	42 (41.6)	*χ*^2^ = 4.92, *p* = 0.03
Disseminating findings to academic audiences (*n* = 243)	60 (36.4)	18 (23.1)	*χ*^2^ = 4.29, *p* = 0.04
The production of useful findings in the field (*n* = 241)	86 (51.2)	29 (39.7)	*χ*^2^ = 2.68, *p* = 0.10
The promotion of evidence-informed decision making in health care or the healthcare system (*n* = 241)	55 (32.7)	20 (27.4)	*χ*^2^ = 0.68, *p* = 0.41
The project’s impact on health care professional practices (*n* = 239)	42 (25.1)	14 (19.4)	*χ*^2^ = 0.91, *p* = 0.34
The project’s impact on health system policies (*n* = 240)	22 (13.2)	8 (11.0)	*χ*^2^ = 0.23, *p* = 0.63

Significant values are shown in bold

Perceived effects of involving knowledge users in the project overall, in specific components of the research process and on project outcomes are reported in Appendix 3. A total of 57% of respondents rated the overall influence of knowledge user involvement in the project as significant, with NPIs having a higher proportion of significant ratings (66%) than other researchers (50%) and knowledge users (51%). Women also reported higher rates of significant ratings (65%) than men (46%).

Ratings of significant influence of knowledge user involvement in individual components of the research process ranged between 32% (dissemination to academic audiences) and 63% (participant recruitment). There were no differences in perceived effects of knowledge user involvement in any of the research components between respondent project roles. There was a significant difference in the proportion of ratings of significant influence of involving knowledge users in data analysis and interpretation between women (43%) and men (19%).

Ratings of significant influence of knowledge user involvement in project outcomes ranged between 12% (project impact on health system policies) and 48% (production of useful research findings). There were no differences in perceived project outcome ratings between respondent project roles or gender.

### Team cohesion (Table 4)

**Table 4:** Perceived team cohesion

Statement	Level of agreement [*n* (%)]				Chi-squared, *p*-value
	Full sample	Project role			
		NPI	Researcher	Knowledge user	
Most members of this team fit what I believe to be the ideal team member (*n* = 536)					
Agree	431 (80.4)	188 (82.5)	159 (76.8)	84 (83.2)	*χ*^2^ = 3.07, *p* = 0.55
Neutral	79 (14.7)	31 (13.6)	36 (17.4)	12 (11.9)	
Disagree	26 (4.9)	9 (3.9)	12 (5.8)	5 (5.0)	
I felt sufficiently included in all activities (*n* = 538)					
Agree	471 (87.5)	214 (93.4)	177 (85.1)	80 (79.2)	***χ***^**2**^** = 22.59**, ***p*** < **0.001**
Neutral	45 (8.4)	12 (5.2)	16 (7.7)	17 (16.8)	
Disagree	22 (4.1)	3 (1.3)	15 (7.2)	4 (4.0)	
Most activities were rewarding (*n* = 537)					
Agree	466 (86.8)	204 (89.5)	177 (85.1)	85 (84.2)	*χ*^2^ = 4.89, *p* = 0.30
Neutral	52 (9.7)	19 (8.3)	20 (9.6)	13 (12.9)	
Disagree	19 (3.5)	5 (2.2)	11 (5.3)	3 (3.0)	
If some members of team decided to leave, I would try to dissuade them (*n* = 530)					
Agree	307 (57.9)	151 (66.8)	99 (48.5)	57 (57.0)	*χ*^2^ = 16.03, *p* < 0.01
Neutral	152 (28.7)	48 (21.2)	72 (35.3)	32 (32.0)	
Disagree	71 (13.4)	27 (11.9)	33 (16.2)	11 (11.0)	
I would participate again with the same team (*n* = 535)					
Agree	411 (76.8)	181 (79.4)	155 (75.2)	75 (74.3)	*χ*^2^ = 2.72, *p* = 0.61
Neutral	76 (14.2)	29 (12.7)	29 (14.1)	18 (17.8)	
Disagree	48 (9.0)	18 (7.9)	22 (10.7)	8 (7.9)	
I liked the research team (*n* = 532)					
Agree	473 (88.9)	206 (90.4)	181 (88.9)	86 (86.9)	*χ*^2^ = 1.72, *p* = 0.79
Neutral	45 (8.5)	18 (7.9)	17 (8.3)	10 (10.1)	
Disagree	14 (2.6)	4 (1.8)	7 (3.4)	3 (3.0)	
I think our team meets frequently enough (*n* = 531)					
Agree	404 (76.1)	183 (80.6)	148 (72.5)	73 (73.0)	*χ*^2^ = 8.60, *p* = 0.07
Neutral	81 (15.3)	25 (11.0)	41 (20.1)	15 (15.0)	
Disagree	46 (8.7)	19 (8.4)	15 (7.4)	12 (12.0)	
Working with this team enables my personal goals for the team (*n* = 533)					
Agree	408 (76.5)	197 (86.8)	143 (69.4)	68 (68.0)	***χ***^**2**^** = 23.92**, ***p*** < **0.001**
Neutral	91 (17.1)	21 (9.3)	45 (21.8)	25 (25.0)	
Disagree	34 (6.4)	9 (4.0)	18 (8.7)	7 (7.0)	
Compared with other teams, this team worked well (*n* = 534)					
Agree	155 (75.2)	196 (86.0)	155 (75.2)	73 (73.0)	*χ*^2^ = 11.99, *p* = 0.02
Neutral	78 (14.6)	20 (8.8)	38 (18.4)	20 (20.0)	
Disagree	32 (6.0)	12 (5.3)	13 (6.3)	7 (7.0)	

Significant values are shown in bold

Agreement with team cohesion statements ranged between 58% (“If some members of the team decided to leave, I would dissuade them”) and 89% (“I liked the research team”). Most team cohesion ratings were not significantly different between groups. The only differences related to the statements “I felt sufficiently included in all activities” and “Working with this team enables my personal goals”, where more NPIs agreed with these statements than researchers or knowledge users. There were no differences in team cohesion ratings between women and men (Appendix 4).

### Capability, opportunity and motivation for working in partnership (Table 5)

**Table 5:** Capability (C), opportunity (O) and motivation (M) for working in partnership

Statement	Level of agreement [*n* (%)]				Chi-squared, *p*-value
	Full sample	Project role			
		NPI	Researcher	Knowledge user	
I have the knowledge and skills to engage (*n* = 537) – C					
Agree	515 (95.9)	220 (95.7)	196 (94.7)	99 (99.0)	*χ*^2^ = 3.54, *p* = 0.47
Neutral	17 (3.2)	8 (3.5)	8 (3.9)	1 (1.0)	
Disagree	5 (0.9)	2 (0.9)	3 (1.4)	0 (0.0)	
I am confident in my ability to engage (*n* = 537) – M					
Agree	501 (93.3)	218 (94.8)	191 (92.3)	92 (92.0)	*χ*^2^ = 3.86, *p* = 0.43
Neutral	30 (5.6)	9 (3.9)	13 (6.3)	8 (8.0)	
Disagree	6 (1.1)	3 (1.3)	3 (1.4)	0 (0.0)	
I have the resources to engage (*n* = 537) – O					
Agree	308 (57.5)	139 (60.4)	116 (56.3)	53 (53.0)	*χ*^2^ = 3.70, *p* = 0.45
Neutral	134 (25.0)	56 (24.3)	48 (23.3)	30 (30.0)	
Disagree	94 (17.5)	35 (15.2)	42 (20.4)	17 (17.0)	
I have support from others to engage (*n* = 537) – O					
Agree	413 (76.9)	181 (78.7)	153 (73.9)	79 (79.0)	*χ*^2^ = 4.70, *p* = 0.32
Neutral	93 (17.3)	35 (15.2)	39 (18.8)	19 (19.0)	
Disagree	31 (5.8)	14 (6.1)	15 (7.2)	2 (2.0)	
There is value in engaging (*n* = 535) – M					
Agree	520 (97.2)	219 (95.6)	203 (98.5)	98 (98.0)	*χ*^2^ = 6.66, *p* = 0.16
Neutral	14 (2.6)	10 (4.4)	2 (1.0)	2 (2.0)	
Disagree	1 (0.2)	0 (0.0)	1 (0.5)	0 (0.0)	
It is my responsibility to engage (*n* = 536) – M					
Agree	486 (90.7)	215 (93.9)	186 (90.7)	85 (85.0)	*χ*^2^ = 9.84, *p* = 0.04
Neutral	43 (8.0)	10 (4.4)	19 (9.2)	14 (14.0)	
Disagree	7 (1.3)	4 (1.7)	2 (1.0)	1 (1.0)	
I intend to engage in the future (*n* = 535) – M					
Agree	491 (91.8)	210 (91.7)	192 (93.2)	89 (89.0)	*χ*^2^ = 3.50, *p* = 0.48
Neutral	34 (6.4)	14 (6.1)	10 (4.9)	10 (10.0)	
Disagree	10 (1.9)	5 (2.2)	4 (1.9)	1 (1.0)	
I feel pressure to engage (*n* = 536) – O					
Agree	156 (29.1)	82 (35.7)	61 (29.5)	13 (13.1)	***χ***^**2**^** = 20.66**, ***p*** < **0.001**
Neutral	136 (25.4)	55 (23.9)	57 (27.5)	24 (24.2)	
Disagree	244 (45.5)	93 (40.4)	89 (43.0)	62 (62.6)	
The decision to engage is beyond my control (*n* = 537) – M					
Agree	48 (8.9)	21 (9.1)	24 (11.6)	3 (3.0)	*χ*^2^ = 10.30, *p* = 0.04
Neutral	106 (19.7)	51 (22.2)	41 (19.8)	14 (14.0)	
Disagree	383 (71.3)	158 (68.7)	142 (68.6)	83 (83.0)	
It is useful to engage (*n* = 537) – M					
Agree	509 (94.8)	217 (94.3)	196 (94.8)	96 (96.0)	*χ*^2^ = 1.32, *p* = 0.86
Neutral	24 (4.5)	12 (5.2)	24 (4.5)	3 (3.0)	
Disagree	4 (0.7)	1 (0.4)	4 (0.7)	1 (1.0)	

Significant values are shown in bold

Statements with the highest rates of agreement related to capability (“I have the knowledge and skills to engage”, 96%) and motivation (“There is value in engaging”, 97%). Most ratings were not significantly different between groups, except that NPIs (36%) and other researchers (30%) agreed with the statement “I feel pressure to engage” more than knowledge users (13%). There were no differences in ratings between women and men (Appendix 5).

## Discussion

In this project, we advance understanding of partnered health research through a comprehensive examination of Canadian practices between 2011 and 2019. Our deliberately broad conceptualization of partnered health research contributes to our ability to assemble the largest and richest dataset on co-produced research to date. This analysis advances the understanding of roles in partnered health research by differentiating between the perceptions of project team members- specifically, between the NPI (the project lead), and other researcher and knowledge user team members. We are also the first to explore the role of gender in team member perceptions. Overall, most of the 589 respondents reported positive perceptions of their own capability, opportunity and motivation to work in health research partnerships and team cohesion on the eligible project, but reported mixed perceptions of the effects of involving knowledge users on the project.

Most perceptions were similar between researcher and knowledge user roles and women and men, with a few noteworthy differences. The positive perceptions described by many respondents are consistent with previous partnership research, including the earlier Canadian study from 2005 to 2010 [12]. Syntheses of factors influencing research partnerships often include more facilitators than barriers [7, 8], and qualitative descriptions from individuals who engage in partnered research often reflect a strong commitment among those involved [38]. The body of evidence continues to support the notion that partnered research is fundamentally a principled approach to science [39].

The majority of respondents (57%) perceived the overall impact of involving knowledge users in the project to be significant. Documenting the effects of partnered research is a known challenge in the field owing to the complex nature of partnering and time lags in uptake and impact. We acknowledge the inherent limitations of assessing perceived effects of partnered research; however, doing so allows comparison across studies. Globally, perceptions on the impact of research involvement are mixed [7, 8]. Respondents in the previous Canadian study indicated very high rates (> 80%) of anticipated impact of partnered research relative to studies that did not involve knowledge users [12]. Most respondents in this study were also positive about the overall effect of involving knowledge users in the project (57% rated the overall influence to be significant). However, when probed about specific effects of involving knowledge users in components of the research process and project impact, respondents were more conservative. Although more than 50% of respondents agreed that the eligible partnered research project would produce useful findings for the field, less than one third agreed that the project would enhance evidence-informed decision-making or influence healthcare professional practice or health policy. Acknowledging that some of these impacts require longer timespans to have effect than the questionnaire accounted for and that it it is possible that not all projects may have had goals to influence these outcomes, these results do suggest that the impacts of knowledge user involvement may be project- and context-specific. While additional study is needed to fully understand these findings, ideally through more in-depth qualitative inquiry, respondent perceptions of capability, opportunity and motivation for working in partnership may offer some clues. For example, the mixed agreement to the statement on having adequate resources to engage (57% agree) suggests ongoing pragmatic challenges to partnering in health research, which can in turn affect outcomes.

Although the similar ratings of most variables suggests that researchers and knowledge users and men and women may be largely aligned in their perspectives, cases of group differences offer some interesting insights. For example, 66% of NPIs reported a significant overall influence of knowledge user involvement in the project compared with 50% of team researchers and 51% of knowledge users. Likewise, NPIs reported higher ratings of agreement for feelings of inclusion, alignment with personal goals and pressure to engage than team researchers or knowledge users. Ratings of reported pressure to engage were also highest among NPIs (and lowest among knowledge users). These findings reflect known tensions in partnered health research. In Canada, the NPI is typically the lead and often the driver of the particular project, and the NPI role may be prioritized by academic institutions tracking funding dollars and valued for academic career advancement considerations [40]. Our finding related to feelings of pressure to engage supports comments by Canadian academic researchers [41] and may be contributing to concerns and experiences of tokenism in engagement expressed by knowledge users [42]. Although the distribution of respondent genders reflected known trends in partnered research dominated by women, most respondent perceptions did not vary between men and women.

We acknowledge the methodological limitations of this study. Similar to other research studying partnered research, we are limited by self-report, respondent bias, indirect assessment of effects and a lack of standardized data collection instruments. Our sample is restricted to a selection of Canadian partnered health research that received project funding. Although we contacted all named principal investigators directly to potentially engage with knowledge users (unlike other approaches that rely on researchers to provide contact information), most survey respondents were researchers, and we did not achieve substantially greater recruitment of knowledge users than recruitment approaches relying on researcher referral. This may have also be related to data collection timing during the coronavirus disease 2019 (COVID-19) pandemic (August 2020). We also recognize that that most of the knowledge users in this sample held a professional role, not people with lived experience of a health condition. This may be because we were relying on publicly available contact information, which would have been more likely for individuals in professional roles. It could also be related to the topics of the research. Recruitment and sampling continue to be challenges in studying partnered research. We were unable to compare more than two genders owing to low cell counts, though we respect that gender is not a binary variable. We acknowledge that the questionnaire did not capture any knowledge user NPIs, which should be addressed in future updates of the instrument. We were unable to analyse or compare project-level variables in this analysis because the incidences of multiple responses for some projects violates assumptions of data independence needed for statistical comparisons. However, the presence of multiple responses for specific projects allows us to directly compare team member responses and examine congruence of perceptions in future analyses. This comprehensive dataset offers rich potential for additional analysis, and we have plans that include integration with qualitative data gathered in follow up and mixed methods approaches (forthcoming publications). These findings can also inform future work to develop evidence-informed practice recommendations for partnered health research.

## Conclusions

Respondents from this large survey of recipients of Canadian partnered health research projects funded between 2011 and 2019 indicated primarily positive perceptions of working in partnership and acknowledged somewhat limited effects of involving knowledge users in specific research components and project impacts. The few group differences observed suggested a distinct perspective among NPIs relative to other researchers and knowledge users involved in the eligible project. These findings highlight the need for continued efforts to explore how to optimize the impact of partnered research and achieve its stated objective. Continued analysis of project level outcomes may identify specific contexts and features associated with greater impact.

## Supplementary Information

Additional file 1.

## Data Availability

The datasets used may be available from the corresponding author on reasonable request.

## Abbreviations

CBPR: Community-based participatory research
POR: Patient-oriented research
IKT: Integrated knowledge translation
CIHR: Canadian Institutes of Health Research
