# Supplementary material for: Partnered health research in Canada: a cross-sectional survey of perceptions among researchers and knowledge users involved in funded projects between 2011 and 2019
Source: Health Res Policy Syst. 2025 Mar 3;23:28. doi: 10.1186/s12961-025-01299-8 (PMC11874841; doi:10.1186/s12961-025-01299-8)
Supplement: Supplementary file 1 — Additional file 1. [file 12961_2025_1299_MOESM1_ESM.docx]

Appendix 1. Questionnaire distributed to participants.

**Health Research Partnerships in Canada Online Questionnaire**

**Section 1: Introduction and Consent**

**Thank you for accessing the Health Research Partnerships in Canada online questionnaire.**

**The purpose of this study is to describe the practices, perceived effects, and perceptions of health research partnerships with researchers and research users in Canada.** In this study, health research partnerships are defined as the involvement of research users (those who are able to use the knowledge generated through research) throughout the research process. Health research partnerships are sometimes referred to as integrated knowledge translation (KT), participatory action research, community-based participatory research, patient and public involvement research, and by other terms. **The findings will increase our understanding of health research partnerships in Canada, and be used to inform the development of health research partnership practice and reporting recommendations.**

**You have been identified as an independent researcher (referred to as researcher) or research user investigator on a Canadian health research project funded between 2011 and 2019 that included a partnership involving research users throughout the research process.** You have been identified through publicly-available information from one of the following health research funding organizations: Canadian Institutes of Health Research (CIHR), Alberta Innovates, Michael Smith Foundation for Health Research, Research Manitoba, Saskatchewan Health Research Foundation. **We value your contributions as a member of a Canadian health research partnership and ask you to participate in this study by completing this questionnaire.**

**The questionnaire takes most people about 20 minutes to complete.** If you agree to complete the questionnaire, the system will automatically save your progress so you can close the questionnaire and return to finish it at a later time. You can return to your place in the survey by clicking on the link in the email that was sent to you.

Participation in this questionnaire is completely voluntary. All participants and research projects will be assigned a unique ID. Your responses will be coded, resulting in the removal of any identifying information, except for some publicly available data such as project name, year funded, funder, and funding program and amount. All information will be kept confidential and will only be used for research purposes. Information from this study may be published and/or shared in public forums, but only in summary forms, and your name and the name of the project will not be used or disclosed. The health research funding organizations will not have access to study data and responses will have no impact on current or future funding decisions. If you would like to receive a summary of findings of this study, please indicate this at the end of the survey.

The risks of participating are low. Participating in this survey will not offer any direct benefits to you, but it will contribute to enhance the understanding of health research partnership practices in Canada. There are no costs to you to participate in this study.

This study is a part of a research program undertaken by a Canadian team based at the University of Manitoba. This study has been funded by the Canadian Institutes of Health Research (Project Grant # PJT-156372, nominated principal investigator: Sibley). CIHR has no role in study design, data collection, analysis, interpretation, reporting or dissemination of results of this study.

The questionnaire is hosted by an online software tool called SurveyMonkey. The privacy policy for this company can be found at <https://www.surveymonkey.com/mp/legal/privacy-policy/>.

This study has been approved by the University of Manitoba Health Research Ethics Board (ethics#). They can be reached at [bannatynereb@umanitoba.ca](mailto:bannatynereb@umanitoba.ca) or 204 789-3255.

The completion of this study requires your free and informed consent. Submitting the questionnaire will imply your free and informed consent and that you understand the above conditions regarding the participation in the study. If you have any questions, please contact Dr. Kathryn Sibley ([Kathryn.sibley@umanitoba.ca](mailto:Kathryn.sibley@umanitoba.ca)).

**Do you consent to participate in this questionnaire?**

- 1. Yes
  2. No 🡪 Skip logic: bring participants to alternate end of survey message.

**Section 2: Eligibility**

Thank you for agreeing to participate in this study. You have been identified for this study because of your role in a Canadian health research project funded between 2011 and 2019 that included a partnership. This section verifies eligibility for this survey.

- 1. You have been identified as part of the project titled [Project Name], which was funded in [Year Funded] by [Funder] for a value of [Value], led by [Nominated Principal Investigator]. Does/did this project involve research users throughout the research process?

*Research users are defined as those who are able to use the knowledge generated through research in order to make informed decisions. Research users include (but are not limited to): people with lived experience of a health condition, communities, community organizations, health professionals, health system decision and policy makers, and health research funding organizations.*

1. Yes
2. No 🡪 Skip logic: bring participants to alternate end of survey message.

Please answer all subsequent questions in relation to this project.

- 1. What is/was your **primary** role in this project?

Project title: [Project Name],

Funded in [Year Funded] by [Funder] for a value of [Value], led by [Nominated PI].

*If you identify as both a researcher and research user, please choose the one which best reflected your contributions to this project.*

- 1. Independent researcher *(i.e., an individual who is autonomous regarding their research activities and/or has an academic or research appointment.* *For the purpose of this study, independent researchers also includes individuals in co-investigator roles and trainee)* 🡪 Skip logic: Include questions 3.1, 9.7; Exclude questions 3.2 and 3.3, and section 8
  2. Research user *(i.e., those who are able to use the knowledge generated through research in order to make informed decisions. Research users include (but are not limited to): people with lived experience of a health condition, communities, community organizations, health professionals, health system decision makers, health policy makers, and health research funding organizations)* 🡪 Skip logic: Include questions 3.2 and 3.3, and section 8; Exclude questions 3.1, 9.7,
  3. Other, please specify 🡪 Skip logic: Include questions 3.2 and 3.3, and section 8; Exclude questions 3.1, 9.7
  4. I don’t know 🡪 Skip logic: bring participants to alternate last page

**Section 3: Role and Project Details**

This section asks more about your role in this project and the current status of this project.

Project title: [Project Name]

Funded in [Year Funded] by [Funder] for a value of [Value], led by [Nominated Principal Investigator].

- 1. Are/were you the **nominated principal investigator** in this project?

*The nominated principal investigator is the individual responsible for coordinating the financial and administrative aspects of the research project, as well as leading the intellectual direction of the proposed activities.*

1. Yes
2. No
3. I don’t know

3.2 What is/was your **primary research user** role in this project?

1. Person with lived experience of a health condition, either directly (*i.e., "patient"*) or indirectly (*i.e., family member, friend, or caregiver*)
2. Community member
3. Health professional
4. Healthcare manager or administrator
5. Health system decision or policy maker
6. Community organization representative
7. Health research funding organization representative

Health professional organization representative

Industry representative

1. Other, please specify:
2. I don’t know 🡪 skip logic: exclude question 3.3

3.3 How many years have you been a/an {{response from #3.2}}?

1. 0-3 years
2. 4-7 years
3. 8-11 years
4. 12 years or more

3.4 What is the current status of this project?

1. Ongoing (*i.e., any research activity or task underway or planned*)🡪 Skip logic: Include questions 3.6, 3.7 and 4.4; 5.2, O5.3-O5.12; 5.24; Exclude question 3.5 and 4.3; 5.1; C5.3-C5.12; 5.13-5.23
2. Completed (*i.e., no further ongoing research activities or tasks*) 🡪 Skip logic: Include question 3.5, 3.7, 4.3; 5.1; C5.3-C5.12; 5.13-5.24; Exclude 3.6; 4.4; 5.2; O5.3-O5.12
3. I don’t know 🡪 Skip logic Include questions 3.6, 3.7 and 4.4; 5.2, O5.3-O5.12; 5.24; Exclude question 3.5 and 4.3; 5.1; C5.3-C5.12; 5.13-5.23

3.5 Which components of the research process were included in this project?

|  | Yes; included in this project **{{**Responses will be carried forward for 4.3}} | No; not included in this project | I don’t know 🡪 Skip logic if this is their only checked answer: exclude question 4.3 and C5.3-C5.12 |
| --- | --- | --- | --- |
| Setting the project’s research priorities |  |  |  |
| Choosing the research questions |  |  |  |
| Development of study design and methods |  |  |  |
| Choosing study outcomes |  |  |  |
| Development of research ethics documents (*e.g., consent forms*) |  |  |  |
| Participant recruitment |  |  |  |
| Data collection |  |  |  |
| Data analysis and interpretation |  |  |  |
| Dissemination (*i.e., sharing*) of research findings to non-academic audiences (*e.g., public presentation, summary of findings*) |  |  |  |
| Dissemination of research findings to academic audiences (*e.g., peer-reviewed journal publication(s)*) |  |  |  |
| Other, please specify: | | | |

3.6 Which components of the research process are/ will be included in this project?

|  | Yes; completed *carry forward for 4.4 | Yes; underway  *carry forward for 4.4 | Yes; planned  *not carried forward | No; not part of this project  *not carried forward | I don’t know  *not carried forward and if this is their only response then skip logic: exclude question 4.4 and C5.3-C5.12 |
| --- | --- | --- | --- | --- | --- |
| Setting the project’s research priorities |  |  |  |  |  |
| Choosing the research questions |  |  |  |  |  |
| Development of study design and methods |  |  |  |  |  |
| Choosing study outcomes |  |  |  |  |  |
| Development of research ethics documents (*e.g., consent forms*) |  |  |  |  |  |
| Participant recruitment |  |  |  |  |  |
| Data collection |  |  |  |  |  |
| Data analysis and interpretation |  |  |  |  |  |
| Dissemination (*i.e., sharing*) of research findings to non-academic audiences (*e.g., public presentation, summary of findings*) |  |  |  |  |  |
| Dissemination of research findings to academic audiences (*e.g., peer-reviewed journal publication(s)*) |  |  |  |  |  |
| Other, please specify: | | | | | |

3.7 Is there anything else you would like to tell us about your role or the current status of this project?

**Section 4: Research Partnership Practices**

This section asks about the features and practices of the research partnership and involvement of research users in this project.

*Health research partnerships are defined as the involvement of research users (those who are able to use the knowledge generated through research) throughout the research process.*

Project title: [Project Name],

Funded in [Year Funded] by [Funder] for a value of [Value], led by [Nominated Principal Investigator].

- 1. What term was used by the research team to refer to the concept of research partnership or involvement of research users in this project? **Check all that apply.**

1. Action Research
2. Community-Based Participatory Research
3. Engaged Scholarship
4. Indigenous or Relational Methodologies
5. Integrated Knowledge Translation
6. Participatory Action Research
7. Participatory Research
8. Patient and Public Involvement
9. No term used for the research partnership
10. Other, please specify:
11. I don’t know

4.2 Which research users are/were involved in this project? **Check all that apply.**

1. Person with lived experience of a health condition, either directly (*i.e., "patient"*) or indirectly (*i.e., family member, friend, or caregiver*)
2. Community member
3. Health professional
4. Healthcare manager or administrator
5. Health system decision or policy maker
6. Community organization representative
7. Health research funding organization representative

Health professional organization representative

Industry representative

1. Other, please specify:
2. I don’t know

4.3 In which components of the research process were research users involved? {{Carry forward function in SM to show only the responses selected by participants}}

|  | Yes *carry forward for section 5 | No  *not carried forward | I don’t know  *not carried forward and Skip logic if this is their only checked answer: exclude C5.3-C5.12 |
| --- | --- | --- | --- |
| Setting the project’s research priorities |  |  |  |
| Choosing the research questions |  |  |  |
| Development of study design and methods |  |  |  |
| Choosing study outcomes |  |  |  |
| Development of research ethics documents (*e.g., consent forms*) |  |  |  |
| Participant recruitment |  |  |  |
| Data collection |  |  |  |
| Data analysis and interpretation |  |  |  |
| Dissemination (*i.e., sharing*) of research findings to non-academic audiences (*e.g., public presentation, summary of findings*) |  |  |  |
| Dissemination of research findings to academic audiences (*e.g., peer-reviewed journal publication(s)*) |  |  |  |

4.4 In which components of the research process are/were research users involved? {{Carry forward function in SM to show only the responses selected by participants}}

|  | Yes *carry forward for section 5 | No  *not carried forward | I don’t know  *not carried forward and Skip logic if this is their only checked answer: exclude O5.3-O5.11 |
| --- | --- | --- | --- |
| Setting the project’s research priorities |  |  |  |
| Choosing the research questions |  |  |  |
| Development of study design and methods |  |  |  |
| Choosing study outcomes |  |  |  |
| Development of research ethics documents (*e.g., consent forms*) |  |  |  |
| Participant recruitment |  |  |  |
| Data collection |  |  |  |
| Data analysis and interpretation |  |  |  |
| Dissemination (*i.e., sharing*) of research findings to non-academic audiences (*e.g., public presentation, summary of findings*) |  |  |  |
| Dissemination of research findings to academic audiences (*e.g., peer-reviewed journal publication(s)*) |  |  |  |

4.5 Is/was the research partnership or involvement of research users informed by a theory, model, and/or framework (e.g. Framework for Collaborative Research, Co-KT Framework, etc.)?

1. Yes, 🡪 Skip logic: Include question 4.5a; 4.6
2. No 🡪 Skip logic: Skip to question 4.7
3. I don’t know 🡪 Skip logic: Skip to question 4.7

4.5a Please specify the theory, model, and/or framework used to inform the research partnership or involvement of research users: ____________________________________________________________________________________________________________________________________________________

4.6 Which components of the research user involvement are/were informed by theory, model and/or framework? **Check all that apply.**

1. Support of researchers’ ability to involve research users and share power in project decision making
2. Support of research users’ ability to be involved and share power in project decision making
3. Maintenance of relationships between researchers and research users
4. Addressing ethical issues
5. Development of funding application
6. Setting the project’s research priorities
7. Choosing the research questions
8. Development of study design and methods
9. Choosing study outcomes
10. Participant recruitment
11. Data collection
12. Data analysis and interpretation
13. Dissemination (*i.e., sharing*) of research findings to non-academic audiences (*e.g., public presentation, summary of findings*)
14. Dissemination of research findings to academic audiences (*e.g., peer-reviewed journal publication(s)*)
15. Evaluation of research user involvement
16. Other, please specify:
17. I don’t know

4.7 Which approaches or activities are /were used for involving research users in this project? **Check all that apply.**

1. Formal in-person research project meeting(s)- individual and/or group
2. Formal online research project meeting(s)- individual and/or group
3. Formal updates or newsletters about the research project- electronic and/or hard copy
4. Informal conversations – in-person, telephone, and/or electronic
5. Distribution of study documents (*e.g., protocols, data collection tools*)
6. Shared electronic space for saving research project documents (*e.g., web portal, password protected site*)
7. Development of formal documentation or process (*e.g., terms of reference, partnership agreements*)
8. Establishment of formal working groups
9. Provision of social opportunities (*e.g., research team dinner*)
10. Provision of training opportunities and/ or resource materials
11. Honorarium for research users
12. Reimbursement of expenses for research users
13. Sharing of research funds with research user organization
14. Researchers attending research user meetings or events *(e.g. invited talks, fundraising events)*
15. Other, please specify:
16. I don’t know

4.8 On average, how often are/were research users involved or included in an activity in this project?

1. 1-4 times a year
2. 5-8 times a year
3. 9-12 times a year

e. Other, please specify:

f. I don’t know

4.9 Was/will the research partnership of this project (be) evaluated?

*Evaluation is defined as the systematic collection of information about the activities, characteristics, and outcomes of program, services, policy, or processes, in order to make judgments about the program/process, improve effectiveness, and/or inform decisions about future development.*

1. Yes 🡪 Skip logic: Include questions 4.10-4.13
2. No 🡪 Skip logic: Skip to question 4.14
3. I don’t know 🡪 Skip logic: Skip to question 4.14

4.10 At what stage of this project was/will the research partnership (be) evaluated? **Check all that apply.**

1. Pre-study
2. During the study
3. Post-study
4. Other, please specify
5. I don’t know

4.11 What type of partnership evaluation of was/will be conducted? **Check all that apply.**

a. Informal evaluation (for internal use only)

b. Formal research study

c. Other, please specify:

d. I don’t know

4.12 What methodologies were/will be used to evaluate the research partnership in this project? **Check all that apply**.

1. Qualitative🡪 Skip logic: Skip to question 4.14
2. Quantitative 🡪 Skip logic: Include question 4.13
3. Mixed methods 🡪 Skip logic: Include question 4.13
4. Other, please specify: 🡪 Skip logic: Skip to question 4.14
5. I don’t know 🡪 Skip logic: Skip to question 4.14

4.13 Was/will any validated instrument (be) used for the evaluation of the research partnership of this project?

1. Yes, 🡪 skip logic: include 4.13a
2. No, 🡪 skip logic: include 4.14
3. I don’t know, 🡪 skip logic: include 4.14

4.13a Please specify the validated instrument that was/will be used for the evaluation of the research partnership of this project: _________________

4.14 Are/ were diversity considerations explicitly incorporated when building the partnership for this project?

*Diversity is defined as the presence of difference, including, but not limited to, differences in race, colour, place of origin, religion, immigrant and newcomer status, ethnic origin, ability, sex, sexual orientation, gender identity, gender expression and age.*

1. Yes 🡪 Skip logic: Include question 4.15
2. No 🡪 Skip logic: Skip to Section 4.16
3. I don’t know 🡪 Skip logic: Skip to Section 4.16

4.15 What type(s) of diversity considerations are/were taken into account? **Check all that apply.**

1. Race *(Race is based primarily upon genetically imparted physiognomical features among which skin colour is a dominant, but not the sole, attribute)*
2. Ethnicity *(Ethnicity includes aspects such as race, origin or ancestry, identity, language and religion*)
3. Indigeneity (*Indigenous peoples is a collective name for the original peoples of North America and their descendants (i.e., First Nations, Inuit and Métis peoples))*
4. Gender
5. Class
6. Sexuality
7. Geography
8. Age
9. Ability
10. Immigration status
11. Religion
12. Other, please specify:
13. I don’t know

4.16 Is there anything else you would like to tell us about the features and practices of the research partnership and involvement of research users in this project?

**Section 5: Perceived Effects of Involving Research Users on the Research Process and Study Outcomes**

This section asks about your views on the effects of involving research users in this project.

Project title: [Project Name]

Funded in [Year Funded] by [Funder] for a value of [Value], led by [Nominated PI].

5.1 In your opinion, how much did involvement of research users in this project influence the overall project? (only “completed” projects will receive this question)

a. No influence

b. A little influence

c. Moderate influence

d. Significant influence

5.2 In your opinion, how much has involvement of research users in this project influenced the overall project to date? (only “ongoing” projects will receive this question)

a. No influence

b. A little influence

c. Moderate influence

d. Significant influence

In your opinion, how much has/did involvement of research users in this project influence(d) the following: (“Ongoing” projects stream)

|  | No influence | A little influence | Moderate influence | Significant influence |
| --- | --- | --- | --- | --- |
| O5.3 Setting the project’s research priorities |  |  |  |  |
| O5.4 Choosing the research questions |  |  |  |  |
| O5.5 Development of study design and methods |  |  |  |  |
| O5.6 Choosing study outcomes |  |  |  |  |
| O5.7 Development of research ethics documents (*e.g., consent forms*) |  |  |  |  |
| O5.8 Participant recruitment |  |  |  |  |
| O5.9 Data collection |  |  |  |  |
| O5.10 Data analysis and interpretation |  |  |  |  |
| O5.11 Dissemination (*i.e., sharing*) of research findings to non-academic audiences (*e.g., public presentation, summary of findings*) |  |  |  |  |
| O5.12 Dissemination of research findings to academic audiences (*e.g., peer-reviewed journal publication(s)*) |  |  |  |  |

In your opinion, how much did involvement of research users in this project influence the following: (“Completed” projects stream)

|  | No influence | A little influence | Moderate influence | Significant influence |
| --- | --- | --- | --- | --- |
| C5.3 Setting the project’s research priorities |  |  |  |  |
| C5.4 Choosing the research questions |  |  |  |  |
| C5.5 Development of study design and methods |  |  |  |  |
| C5.6 Choosing study outcomes |  |  |  |  |
| C5.7 Development of research ethics documents (*e.g., consent forms*) |  |  |  |  |
| C5.8 Participant recruitment |  |  |  |  |
| C5.9 Data collection |  |  |  |  |
| C5.10 Data analysis and interpretation |  |  |  |  |
| C5.11 Dissemination (*i.e., sharing*) of research findings to non-academic audiences (*e.g., public presentation, summary of findings*) |  |  |  |  |
| C5.12 Dissemination of research findings to academic audiences (*e.g., peer-reviewed journal publication(s)*) |  |  |  |  |

(Only “completed” projects will receive 5.13-5.23)

5.13 In your opinion, to what extent did this project influence **research user**  knowledge and/or skills for engaging in research activities?

1. No influence
2. A little influence
3. Moderate influence
4. Significant influence

5.14 In your opinion, to what extent did this project influence **researcher** knowledge and/or skills for involving research users in research partnerships?

1. No influence
2. A little influence
3. Moderate influence
4. Significant influence

5.15 In your opinion, to what extent did this project produce useful research findings for the research users on the team?

1. No influence
2. A little influence
3. Moderate influence
4. Significant influence

5.16 In your opinion, to what extent did the project findings influence a specific decision by research users on the team?

1. No influence
2. A little influence
3. Moderate influence
4. Significant influence

5.17 In your opinion, to what extent did the project findings influence a specific action by research users on the team?

1. No influence
2. A little influence
3. Moderate influence
4. Significant influence

5.18 In your opinion, to what extent did this project influence evidence-informed decision making by research users on the team?

1. No influence
2. A little influence
3. Moderate influence
4. Significant influence

In your opinion, to what extent do you believe involvement of research users in this project influence the following:

|  | No influence | A little influence | Moderate influence | Significant influence |
| --- | --- | --- | --- | --- |
| 5.19 The production of useful research findings in the field |  |  |  |  |
| 5.20 The promotion of evidence-informed decision making in health care or the health system |  |  |  |  |
| 5.21 The projects’ impact on health care professional practices |  |  |  |  |
| 5.22 The project’s impact on health system policies |  |  |  |  |
| 5.23 Other, please specify: | | | | |

5.24 Is there anything else you would like to tell us about your views on the effects of involving research users in this project? (both ongoing and completed projects will get this question)

**Section 6: Teamwork in the Research Partnership**

This section asks about your experience and views as a team member working on this project. This will help us understand relationships between how teams work together and partnership processes and study outcomes.

Project title: [Project Name]

Funded in [Year Funded] by [Funder] for a value of [Value], led by [Nominated PI].

Please indicate your level of agreement with each statement for this project.

|  | Strongly disagree | Disagree | Neutral | Agree | Strongly agree |
| --- | --- | --- | --- | --- | --- |
| 6.1 Most members of this research team fit what I believe to be the ideal team member |  |  |  |  |  |
| 6.2 I feel that I am/was sufficiently included by the research team in all the team’s activities |  |  |  |  |  |
| 6.3 I find/found most of the activities in which I participate/ participated as a member of this team rewarding |  |  |  |  |  |
| 6.4 If some members of the research team decided to dissolve the team by leaving, I would (have) try/tried to dissuade them |  |  |  |  |  |
| 6.5 If asked to participate in another project like this one, I would like to be with the same people who are/were involved in this project |  |  |  |  |  |
| 6.6 I like/liked the research team I am/was working with |  |  |  |  |  |
| 6.7 I think our research team meets/met frequently enough |  |  |  |  |  |
| 6.8 I feel that working with this particular research team enables/ enabled me to attain my personal goals for which the team was formed |  |  |  |  |  |
| 6.9 Compared to other research teams, my team works/ worked well together |  |  |  |  |  |

6.10 Is there anything else you would like to tell us about your views on how researchers and research users worked together as a team for this project?

**Section 7: Working in Research Partnerships**

This section asks about your views on your ability to be involved in research partnerships. This information will help us understand relationships between these factors and effects on partnership processes and study outcomes.

Please indicate your level of agreement with each statement for this project.

|  | Strongly disagree | Disagree | Neutral | Agree | Strongly agree |
| --- | --- | --- | --- | --- | --- |
| 7.1 I have the knowledge and skills to engage in research partnerships |  |  |  |  |  |
| 7.2 I am confident in my ability to engage in research partnerships |  |  |  |  |  |
| 7.3 I have the resources (*e.g., time and money)* to engage in research partnerships |  |  |  |  |  |
| 7.4 I have support from others (*e.g., colleagues, friends, own organization, etc.)* to engage in research partnerships |  |  |  |  |  |
| 7.5 There is value in engaging in research partnerships |  |  |  |  |  |
| 7.6 It is my responsibility to engage in research partnerships |  |  |  |  |  |
| 7.7 I intend to engage in research partnerships in future |  |  |  |  |  |
| 7.8 I feel pressure to engage in research partnerships |  |  |  |  |  |
| 7.9 The decision to engage in research partnerships is beyond my control |  |  |  |  |  |
| 7.10 It is useful to engage in research partnerships |  |  |  |  |  |

7.11 Is there anything else you would like to tell us about your views on your experience working in research partnerships?

**Section 8: Research User Experiences**

*Skip logic- this section is to be filled out by research users only.*

This section asks about your general experiences as a research user throughout this project.

Project title: [Project Name]

[Year Funded] by [Funder] for a value of [Value], led by [Nominated PI].

Please rate your level of agreement with each statement for this project.

|  | Strongly disagree | Disagree | Neutral | Agree | Strongly agree |
| --- | --- | --- | --- | --- | --- |
| 8.1 I feel/felt comfortable contributing my knowledge, experience, preferences, values or opinions in this project |  |  |  |  |  |
| 8.2 I believe that my contributions are/were valued or appreciated in this project |  |  |  |  |  |
| 8.3 I believe the researchers take/took my participation in this project seriously |  |  |  |  |  |
| 8.4 I believe that my input influences/influenced final decisions in this project |  |  |  |  |  |
| 8.5 The researchers are/were neutral in their opinions during this project |  |  |  |  |  |
| 8.6 I clearly understand/ understood my role in this project |  |  |  |  |  |
| 8.7 All research partnership members have/had equal opportunity to contribute to decision making in this project |  |  |  |  |  |
| 8.8 Information is/was made available to me either prior to or during the research project activities so I can/could participate knowledgeably in this project |  |  |  |  |  |
| 8.9 The information provided to me during this project is/was easy to understand |  |  |  |  |  |
| 8.10 I clearly understand/ understood what is/was expected of me in this project |  |  |  |  |  |
| 8.11 I clearly understand/ understood the goals of this project |  |  |  |  |  |
| 8.12 I would/did use the findings of this project |  |  |  |  |  |

8.13 Is there anything else you would like to tell us about your general experiences as a research user throughout this project?

**Section 9: Respondent Characteristics**

This section asks about you.

9.1 Please indicate the gender you identify with:

*Gender is defined as a person's sense of being a woman, man, both, neither, or anywhere along the gender spectrum. A person's gender identity may be the same as, or different, from their birth-assigned sex.*

1. Prefer not to answer
2. The gender I identify with is: ______

9.2 Do you identify as a member of a visible minority in Canada?

*Visible minority refers to whether a person belongs to a visible minority group as defined by the Employment Equity Act which defines visible minorities as persons, other than Aboriginal peoples, who are non-Caucasian in race or non-white in colour.*

1. Yes
2. No
3. Prefer not to answer

9.3 Do you identify as Indigenous?

1. Yes 🡪 Skip logic: Include questions 9.4
2. No 🡪 Skip logic: Skip to question 9.5
3. Prefer not to answer 🡪 Skip logic: Skip to question 9.5

9.4 Please select the option that you identify with:

1. First Nations🡪 Skip logic: include question 9.4a
2. Inuk (Inuit)
3. Métis
4. Prefer not to answer

9.4a Please specify the First Nations that you identify with:

____________________________________________________________________________________________________________________________________________________________

9.5 How many research partnerships have you been involved in?

1. 1-3
2. 4-6
3. 7 or more

9.6 Do you have any formal training in research partnerships?

1. Yes 🡪 Skip logic: include 9.6a
2. No

9.6a Please describe the training you received in research partnerships: ____________________________________________________________________________________________________________________________________________________________

9.7 At the time this grant was awarded [year of funding], how many years had you been an independent researcher?

1. 0-4 Years
2. 5-15 Years
3. More than 15 years
4. I don’t know

9.8 Is there anything else you would like to tell us about yourself?

**…**

Thank you for completing this questionnaire! Your responses are highly appreciated and will help to advance understanding of health research partnerships in Canada.

We plan to conduct follow-up interviews with a sample of survey participants to explore partnership experiences in greater depth. **Would you be willing to be contacted to receive more information on the interview study?**

Clicking "Yes" does not imply that you consent to participate in subsequent research initiatives, it simply authorizes us to contact you.

1. Yes
2. No

…

**Summary of Findings**

Would you like to receive a summary of the findings of this study?

1. Yes
2. No

**…**

**End of survey message:**

The survey is completed. Thank you for your participation and for the information you shared with us. What you shared will help us understand the practices, perceived effects, and perceptions of health research partnerships with researchers and research users in Canada.

We would like to remind you that all information that you have provided us will be kept completely confidential.

If you have any questions, comments, or concerns we would be happy to speak with you. You may contact Dr. Kathryn Sibley at [Kathryn.sibley@umanitoba.ca](mailto:Kathryn.sibley@umanitoba.ca).

Thank you again for your time.

**…**

**Alternative end of survey message (no consent/ineligible):**

Based on your answers, you have been brought to the end of the survey. If you believe this is a mistake, please click on the previous button to review your responses. Thank you for your participation and for the information you shared with us.

We would like to remind you that all information that you have provided us will be kept confidential.

If you have any questions, comments, or concerns we would be happy to speak with you. You may contact Dr. Kathryn Sibley at [Kathryn.sibley@umanitoba.ca](mailto:Kathryn.sibley@umanitoba.ca).

Thank you again for your time.

Appendix 2. **Questionnaire and Derived Variables**

| **Section** | **Question** | **Variable** | **Type** | **Levels/ coding** | **Data organization** | **Analysis** | **Denominator** | **Merged/ Derived variables** |
| --- | --- | --- | --- | --- | --- | --- | --- | --- |
| 1: Introduction and Consent | Do you consent to participate in this questionnaire? | Consent | Dichotomous; 2 levels | 0= No  1= Yes | - 0’s don’t complete survey | - Frequencies: - 0’s = declined - 1’s = consented | N= Returned questionnaires (0’s + 1’s) | / |
| 2: Eligibility | 2.1 You have been identified as part of the project titled [Project Name] which was funded in [Year Funded] by [Funder] for a value of [Value], led by [Nominated Principal Investigator], Does/did this project involve research users in the research process? | Eligibility | Dichotomous; 2 levels | 0= No  1= Yes | - 0’s don’t complete survey | Frequencies:   - 0’s = ineligible n - 1’s = eligible n* | N= Consented | / |
| 2: Eligibility | 2.2 What is/was your primary role in this project? | Role | Nominal; 4 levels | 0= I don’t know  1= Independent researcher  2= Research User  3= Other | - 0’s don’t complete survey - 3’s get coded | Frequencies:   - 1’s = independent researchers - 2’s = research users | N = Eligible (2.1) | Merged with coded 3’s (if possible) |
| 2: Eligibility | 2.2 Other specification | Other_specify | Open ended | Responses will be coded into the available response options or coded into new categories when appropriate. If the response is unclear or there is a small number (<5) of responses in a given category, the responses will be kept as “other”. | | | N = Eligible (2.1) | Merged with 2.2 responses (if possible) |
| 3: Role and Project Details | 3.1 Are/were you the nominated principal investigator in this project? | Researcher_  role_specify | Nominal; 3 levels | 0= I don’t know  1= Yes  2= No | - 1’s definitive answer if multiple researchers answer for one project) | Frequencies:   - 1’s = NPIs - 2’s = Not NPIs | N = Researchers (2.2) | / |
| 3: Role and Project Details | 3.2 What is/was your primary research user role in this project? | Research user_  role_specify | Nominal; 11 levels | 0= I don’t know  1= Person with lived experience of a health condition,  2= Community member  3= Health professional  4= Healthcare manager or administrator  5= Health system decision or policy maker  6= Community organization representative  7= Health research funding organization representative  8= Health professional organization representative  9= Industry representative  10= Other | - 10’s get coded | Frequencies (1-10) | N = Researcher Users (2.2) | Merged with coded 10’s (if possible) |
| 3: Role and Project Details | 3.2 Other specification | Other_specify | Open-ended | Responses will be coded into the available response options or coded into new categories when appropriate. If the response is unclear or there is a small number (<5) of responses in a given category, the responses will be kept as “other”. | | | N = Researcher Users (2.2) | Merged with 3.2 responses (if possible) |
| 3: Role and Project Details | 3.3 How many years have you been a/an {{response from #3.2}}? | Years_  experience_RU | Ordinal; 4 levels | 1= 0-3 years  2= 4-7 years  3= 8-11 years  4= 12 years or more | / | Frequencies | N = Researcher Users (2.2) – 0’s from 3.2 | / |
| 3: Role and Project Details | 3.4 What is the current status of this project? | Project_status | Nominal; 3 levels | 0= I don’t know  1= Ongoing  2=Completed | 0’s merged with 1’s (they receive the same skip logic in the survey) | Frequencies | N = Eligible (2.1) | / |
| 3: Role and Project Details | 3.5 Which components of the research process were included in this project? {{Responses will be carried forward for 4.3}}  3.5a Setting the project’s research priorities | Components_  included_completed  Completed_  components_priorities | Nominal; 3 levels | 0= I don’t know  1= Yes  2= No | 0’s and 2’s removed from analysis | Frequencies | N= Completed projects (2’s from 3.4) | Merged with 1’s and 2’s from 3.6a—see derived table |
| 3: Role and Project Details | 3.5b Choosing the research questions | Completed_  Completed _questions | Nominal; 3 levels | 0= I don’t know  1= Yes  2= No | 0’s and 2’s removed from analysis | Frequencies | N= Completed projects (2’s from 3.4) | Merged with 1’s and 2’s from 3.6b—see derived table |
| 3: Role and Project Details | 3.5c Development of study design and methods | Completed _  components_methods | Nominal; 3 levels | 0= I don’t know  1= Yes  2= No | 0’s and 2’s removed from analysis | Frequencies | N= Completed projects (2’s from 3.4) | Merged with 1’s and 2’s from 3.6c —see derived table |
|  | 3.5d Choosing study outcomes | Completed_components_outcomes | Nominal; 3 levels | 0= I don’t know  1= Yes  2= No | 0’s and 2’s removed from analysis | Frequencies | N= Completed projects (2’s from 3.4) | Merged with 1’s and 2’s from 3.6d —see derived table |
| 3: Role and Project Details | 3.5e Development of research ethics documents (e.g., consent forms) | Completed _  components_ethics | Nominal; 3 levels | 0= I don’t know  1= Yes  2= No | 0’s and 2’s removed from analysis | Frequencies | N= Completed projects (2’s from 3.4) | Merged with 1’s and 2’s from 3.6e —see derived table |
| 3: Role and Project Details | 3.5f Participant recruitment | Completed _  components_recruit | Nominal; 3 levels | 0= I don’t know  1= Yes  2= No | 0’s and 2’s removed from analysis | Frequencies | N= Completed projects (2’s from 3.4) | Merged with 1’s and 2’s from 3.6f —see derived table |
| 3: Role and Project Details | 3.5g Data collection | Completed _  components_data collection | Nominal; 3 levels | 0= I don’t know  1= Yes  2= No | 0’s and 2’s removed from analysis | Frequencies | N= Completed projects (2’s from 3.4) | Merged with 1’s and 2’s from 3.6g —see derived table |
| 3: Role and Project Details | 3.5h Data analysis and interpretation | Completed _  components_analysis | Nominal; 3 levels | 0= I don’t know  1= Yes  2= No | 0’s and 2’s removed from analysis | Frequencies | N= Completed projects (2’s from 3.4) | Merged with 1’s and 2’s from 3.6h —see derived table |
| 3: Role and Project Details | 3.5i Dissemination (i.e., sharing) of research findings to non-academic audiences | Completed _  components_dissem  _nonacademics | Nominal; 3 levels | 0= I don’t know  1= Yes  2= No | 0’s and 2’s removed from analysis | Frequencies | N= Completed projects (2’s from 3.4) | Merged with 1’s and 2’s from 3.6i —see derived table |
| 3: Role and Project Details | 3.5j Dissemination of research findings to academic audiences | Completed _  components_dissem  _academics | Nominal; 3 levels | 0= I don’t know  1= Yes  2= No | 0’s and 2’s removed from analysis | Frequencies | N= Completed projects (2’s from 3.4) | Merged with 1’s and 2’s from 3.6j —see derived table |
| 3: Role and Project Details | 3.5k Other, specify: | Completed_ components_ other | Open ended | Responses will be coded into the available response options or coded into new categories when appropriate. If the response is unclear or there is a small number (<5) of responses in a given category, the responses will be kept as “other”. | | | N= Completed projects (2’s from 3.4) | Merged with 3.5 response options if possible |
| 3: Role and Project Details | 3.6 Which components of the research process are/ will be included in this project? Check all that apply.  3.6a Setting the project’s research priorities | Components_  included_ongoing  Ongoing_  components_priorities | Nominal; 5 levels | 0= I don’t know  1= Yes; completed  2= Yes; underway  3= Yes; planned  4=No; not part of this project | - 1’s + 2’s combined - 0’s, 3’s, and 4’s removed from analysis | Frequencies | N= Ongoing projects (1’s + 0’s from 3.4) | Merge 1’s and 2’s with 3.5a —see derived table |
| 3: Role and Project Details | 3.6b Choosing research questions | Ongoing_  components_questions | Nominal; 5 levels | 0= I don’t know  1= Yes; completed  2= Yes; underway  3= Yes; planned  4= No; not part of this project | - 1’s + 2’s combined - 0’s, 3’s, and 4’s removed from analysis | Frequencies | N= Ongoing projects (1’s + 0’s from 3.4) | Merge 1’s and 2’s with 3.5b —see derived table |
| 3: Role and Project Details | 3.6c Development of study design and methods | Ongoing_  components_methods | Nominal; 5 levels | 0= I don’t know  1= Yes; completed  2= Yes; underway  3= Yes; planned  4= No; not part of this project | - 1’s + 2’s combined - 0’s, 3’s, and 4’s removed from analysis | Frequencies | N= Ongoing projects (1’s + 0’s from 3.4) | Merge 1’s and 2’s with 3.5c —see derived table |
| 3: Role and Project Details | 3.6d Choosing study outcomes | Ongoing_  components_outcomes | Nominal; 5 levels | 0= I don’t know  1= Yes; completed  2= Yes; underway  3= Yes; planned  4= No; not part of this project | - 1’s + 2’s combined - 0’s, 3’s, and 4’s removed from analysis | Frequencies | N= Ongoing projects (1’s + 0’s from 3.4) | Merge 1’s and 2’s with 3.5d —see derived table |
| 3: Role and Project Details | 3.6e Development of research ethics documents (e.g., consent forms) | Ongoing_  components_ethics | Nominal; 5 levels | 0= I don’t know  1= Yes; completed  2= Yes; underway  3= Yes; planned  4= No; not part of this project | - 1’s + 2’s combined - 0’s, 3’s, and 4’s removed from analysis | Frequencies | N= Ongoing projects (1’s + 0’s from 3.4) | Merge 1’s and 2’s with 3.5e —see derived table |
| 3: Role and Project Details | 3.6f Participant recruitment | Ongoing_  components_recruit | Nominal; 5 levels | 0= I don’t know  1= Yes; completed  2= Yes; underway  3= Yes; planned  4= No; not part of this project | - 1’s + 2’s combined - 0’s, 3’s, and 4’s removed from analysis | Frequencies | N= Ongoing projects (1’s + 0’s from 3.4) | Merge 1’s and 2’s with 3.5f —see derived table |
| 3: Role and Project Details | 3.6g Data collection | Ongoing_  components_data collection | Nominal; 5 levels | 0= I don’t know  1= Yes; completed  2= Yes; underway  3= Yes; planned  4= No; not part of this project | - 1’s + 2’s combined - 0’s, 3’s, and 4’s removed from analysis | Frequencies | N= Ongoing projects (1’s + 0’s from 3.4) | Merge 1’s and 2’s with 3.5g —see derived table |
| Section 3: Role and Project Details | 3.6h Data analysis and interpretation | Ongoing_  components_analysis | Nominal; 5 levels | 0= I don’t know  1= Yes; completed  2= Yes; underway  3= Yes; planned  4= No; not part of this project | - 1’s + 2’s combined - 0’s, 3’s, and 4’s removed from analysis | Frequencies | N= Ongoing projects (1’s + 0’s from 3.4) | Merge 1’s and 2’s with 3.5h —see derived table |
| Section 3: Role and Project Details | 3.6i Dissemination (i.e., sharing) of research findings to non-academic audiences | Ongoing_  components_dissem  _nonacademics | Nominal; 5 levels | 0= I don’t know  1= Yes; completed  2= Yes; underway  3= Yes; planned  4= No; not part of this project | - 1’s + 2’s combined - 0’s, 3’s, and 4’s removed from analysis | Frequencies | N= Ongoing projects (1’s + 0’s from 3.4) | Merge 1’s and 2’s with 3.5i —see derived table |
| 3: Role and Project Details | 3.6j Dissemination of research findings to academic audiences | Ongoing_  components_dissem  _academics | Nominal; 5 levels | 0= I don’t know  1= Yes; completed  2= Yes; underway  3= Yes; planned  4= No; not part of this project | - 1’s + 2’s combined - 0’s, 3’s, and 4’s removed from analysis | Frequencies | N= Ongoing projects (1’s + 0’s from 3.4) | Merge 1’s and 2’s with 3.5j —see derived table |
| 3: Role and Project Details | 3.6k Other, please specify | Ongoing_componenets_ other | Open ended | Responses will be coded into the available response options or coded into new categories when appropriate. If the response is unclear or there is a small number (<5) of responses in a given category, the responses will be kept as “other”. | | | N= Ongoing projects (1’s + 0’s from 3.4) | Merge with 3.6 response options if possible |
| 3: Role and Project Details | 3.7 Is there anything else you would like to tell us about your role or the current status of this project? | Ongoing and completed_other | Open ended | Responses will be coded into categories when appropriate. | | | N = Eligible (2.1) | Merge with 3.6 response options if possible |
| 4: Research Partnership Practices | 4.1 What term was used by the research team to refer to the concept of research partnership or involvement of research users in this project? Check all that apply.  4.1a. Action Research | PD_terms  PD_terms_  AR | Dichotomous | 1= Selected  2= Not selected | 2’s removed from analysis | Frequencies | N = Eligible (2.1) | / |
| 4: Research Partnership Practices | 4.1b Community-Based Participatory Research | PD_terms_  CBPR | Dichotomous | 1= Selected  2= Not selected | 2’s removed from analysis | Frequencies | N = Eligible (2.1) | / |
| 4: Research Partnership Practices | 4.1c Engaged Scholarship | PD_terms_  ES | Dichotomous | 1= Selected  2= Not selected | 2’s removed from analysis | Frequencies | N = Eligible (2.1) | / |
| 4: Research Partnership Practices | 4.1d Indigenous or Relational Methodologies | PD_terms_  IRM | Dichotomous | 1= Selected  2= Not selected | 2’s removed from analysis | Frequencies | N = Eligible (2.1) | / |
| 4: Research Partnership Practices | 4.1e Integrated Knowledge Translation | PD_terms_  iKT | Dichotomous | 1= Selected  2= Not selected | 2’s removed from analysis | Frequencies | N = Eligible (2.1) | / |
| 4: Research Partnership Practices | 4.1f Participatory Action Research | PD_terms_  PA | Dichotomous | 1= Selected  2= Not selected | 2’s removed from analysis | Frequencies | N = Eligible (2.1) | / |
| 4: Research Partnership Practices | 4.1g Participatory Research | PD_terms_  PR | Dichotomous | 1= Selected  2= Not selected | 2’s removed from analysis | Frequencies | N = Eligible (2.1) | / |
| 4: Research Partnership Practices | 4.1h Patient and Public Involvement | PD_terms_  PPI | Dichotomous | 1= Selected  2= Not selected | 2’s removed from analysis | Frequencies | N = Eligible (2.1) | / |
| 4: Research Partnership Practices | 4.1i No term used for the research partnership | PD_terms_  No terms | Dichotomous | 1= Selected  2= Not selected | 2’s removed from analysis | Frequencies | N = Eligible (2.1) | / |
| 4: Research Partnership Practices | 4.1j Other, please specify: | PD_terms_  other | Dichotomous | 1= Selected  2= Not selected | 2’s removed from analysis  1’s coded | Frequencies | N = Eligible (2.1) | / |
| 4: Research Partnership Practices | 4.1k I don’t know | PD_terms_  IDK | Dichotomous | 1= Selected  2= Not selected | 2’s removed from analysis | Frequencies | N = Eligible (2.1) | / |
| 4: Research Partnership Practices | 4.1j Other specification | PD_terms_  Specified | Open-ended | Responses will be coded into the available response options or coded into new categories when appropriate. If the response is unclear or there is a small number (<5) of responses in a given category, the responses will be kept as “other”. | | | N = Eligible (2.1) | Merged with 4.1 if possible |
| 4: Research Partnership Practices | 4.2 Which research users are/were involved in this project? Check all that apply.  4.2a Person with lived experience of a health condition | RU_type  RUs_PWLE | Dichotomous | 1= Selected  2= Not selected | 2’s removed from analysis  1’s coded | Frequencies | N = Eligible (2.1) | / |
| 4: Research Partnership Practices | 4.2b Community member | RUs_com member | Dichotomous | 1= Selected  2= Not selected | 2’s removed from analysis  1’s coded | Frequencies | N = Eligible (2.1) | / |
| 4: Research Partnership Practices | 4.2c Health professional | RUs_health prof | Dichotomous | 1= Selected  2= Not selected | 2’s removed from analysis  1’s coded | Frequencies | N = Eligible (2.1) | / |
| 4: Research Partnership Practices | 4.2d Healthcare manager or administrated | RUs_health manager | Dichotomous | 1= Selected  2= Not selected | 2’s removed from analysis  1’s coded | Frequencies | N = Eligible (2.1) | / |
| 4: Research Partnership Practices | 4.2e Health system decision or policy maker | RUs_policy | Dichotomous | 1= Selected  2= Not selected | 2’s removed from analysis  1’s coded | Frequencies | N = Eligible (2.1) | / |
| 4: Research Partnership Practices | 4.2f Community organization representative | RUs_com org | Dichotomous | 1= Selected  2= Not selected | 2’s removed from analysis  1’s coded | Frequencies | N = Eligible (2.1) | / |
| 4: Research Partnership Practices | 4.2g Health research funding organization representative | RUs_funding org | Dichotomous | 1= Selected  2= Not selected | 2’s removed from analysis  1’s coded | Frequencies | N = Eligible (2.1) | / |
| 4: Research Partnership Practices | 4.2h Health professional organization representative | RUs_health prof org | Dichotomous | 1= Selected  2= Not selected | 2’s removed from analysis  1’s coded | Frequencies | N = Eligible (2.1) | / |
| 4: Research Partnership Practices | 4.2i Industry representative | RUs_industry | Dichotomous | 1= Selected  2= Not selected | 2’s removed from analysis  1’s coded | Frequencies | N = Eligible (2.1) | / |
| 4: Research Partnership Practices | 4.2j Other, please specify | RUs_other | Dichotomous | 1= Selected  2= Not selected | 2’s removed from analysis  1’s coded | Frequencies | N = Eligible (2.1) | / |
| 4: Research Partnership Practices | 4.2k I don’t know | RUs_IDK | Dichotomous | 1= Selected  2= Not selected | 2’s removed from analysis  1’s coded | Frequencies | N = Eligible (2.1) | / |
| 4: Research Partnership Practices | 4.2j Other specification | RUs_other specification | Open-ended | Responses will be coded into the available response options or coded into new categories when appropriate. If the response is unclear or there is a small number (<5) of responses in a given category, the responses will be kept as “other”. | | | N = Eligible (2.1) | Merged with 4.2 if possible |
| 4: Research Partnership Practices | 4.3 In which components of the research process were research users involved? {{Carry forward function in SM to show only the responses selected by participants}}  4.3a Setting the project’s research priorities | Completed_RUs  Phases  Completed_RU  _priorities | Nominal; 3 levels | 0= I don’t know  1= Yes  2= No | 0’s and 2’s removed from analysis | Frequencies | N= 1’s from Completed_  components  _priorities (3.5a) | Merged with 1’s from 4.4a—see derived table |
| 4: Research Partnership Practices | 4.3b Choosing the research questions | Completed_  RUs _questions | Nominal; 3 levels | 0= I don’t know  1= Yes  2= No | 0’s and 2’s removed from analysis | Frequencies | N= 1’s from Completed_  components  _questions (3.5b) | Merged with 1’s from 4.4b—see derived table |
| 4: Research Partnership Practices | 4.3c Development of study design and methods | Completed _  RUs_methods | Nominal; 3 levels | 0= I don’t know  1= Yes  2= No | 0’s and 2’s removed from analysis | Frequencies | N= 1’s from Completed_  components  _methods (3.5c) | Merged with 1’s from 4.4c —see derived table |
| 4: Research Partnership Practices | 4.3d Choosing study outcomes | Completed_  RUs outcomes | Nominal; 3 levels | 0= I don’t know  1= Yes  2= No | 0’s and 2’s removed from analysis | Frequencies | N= 1’s from Completed_  components  _questions (3.5d) | Merged with 1’s from 4.4d—see derived table |
| 4: Research Partnership Practices | 4.3e Development of research ethics documents (e.g., consent forms) | Completed _  RUs_ethics | Nominal; 3 levels | 0= I don’t know  1= Yes  2= No | 0’s and 2’s removed from analysis | Frequencies | N= 1’s from Completed_  components  _ethics (3.5e) | Merged with 1’s from 4.4e —see derived table |
| 4: Research Partnership Practices | 4.3f Participant recruitment | Completed _  RUs_recruit | Nominal; 3 levels | 0= I don’t know  1= Yes  2= No | 0’s and 2’s removed from analysis | Frequencies | N= 1’s from Completed_  components  _recruit (3.5f) | Merged with 1’s from 4.4f—see derived table |
| 4: Research Partnership Practices | 4.3g Data collection | Completed _  RUs_data collection | Nominal; 3 levels | 0= I don’t know  1= Yes  2= No | 0’s and 2’s removed from analysis | Frequencies | N= 1’s from Completed_  components  _data collection (3.5g) | Merged with 1’s from 4.4g —see derived table |
| 4: Research Partnership Practices | 4.3h Data analysis and interpretation | Completed _  RUs_analysis | Nominal; 3 levels | 0= I don’t know  1= Yes  2= No | 0’s and 2’s removed from analysis | Frequencies | N= 1’s from Completed_  components  _analysis (3.5h) | Merged with 1’s from 4.4h —see derived table |
| 4: Research Partnership Practices | 4.3i Dissemination (i.e., sharing) of research findings to non-academic audiences | Completed _  RUs_dissem  _nonacademics | Nominal; 3 levels | 0= I don’t know  1= Yes  2= No | 0’s and 2’s removed from analysis | Frequencies | N= 1’s from Completed_  components  _dissem  _nonacademics (3.5i) | Merged with 1’s from 4.4i —see derived table |
| 4: Research Partnership Practices | 4.3j Dissemination of research findings to academic audiences | Completed _  RUs_dissem  _academics | Nominal; 3 levels | 0= I don’t know  1= Yes  2= No | 0’s and 2’s removed from analysis | Frequencies | N= 1’s from Completed_  components  _dissem  _academics (3.5j) | Merged with 1’s from 4.4j —see derived table |
| 4: Research Partnership Practices | 4.4 In which components of the research process are/were research users involved? {{Carry forward function in SM to show only the responses selected by participants}}  4.4a Setting the project’s research priorities | Ongoing_RUs  Phases  Ongoing_RU  _priorities | Nominal; 3 levels | 0= I don’t know  1= Yes  2= No | 0’s and 2’s removed from analysis | Frequencies | N= 1’s and 2’s from Ongoing_  components  _priorities (3.6a) | Merged with 1’s from 4.3a—see derived table |
| 4: Research Partnership Practices | 4.4b Choosing the research questions | Ongoing _  RUs _questions | Nominal; 3 levels | 0= I don’t know  1= Yes  2= No | 0’s and 2’s removed from analysis | Frequencies | N= 1’s and 2’s from Ongoing_  components  _questions (3.6b) | Merged with 1’s from 4.3b—see derived table |
| 4: Research Partnership Practices | 4.4c Development of study design and methods | Ongoing _  RUs_methods | Nominal; 3 levels | 0= I don’t know  1= Yes  2= No | 0’s and 2’s removed from analysis | Frequencies | N= 1’s and 2’s from Ongoing_  components  _methods (3.6d) | Merged with 1’s from 4.3c —see derived table |
| 4: Research Partnership Practices | 4.4d Choosing study outcomes | Ongoing _  RUs _outcomes | Nominal; 3 levels | 0= I don’t know  1= Yes  2= No | 0’s and 2’s removed from analysis | Frequencies | N= 1’s and 2’s from Ongoing_  components  _questions (3.6c) | Merged with 1’s from 4.3d—see derived table |
| 4: Research Partnership Practices | 4.4e Development of research ethics documents (e.g., consent forms) | Ongoing _  RUs_ethics | Nominal; 3 levels | 0= I don’t know  1= Yes  2= No | 0’s and 2’s removed from analysis | Frequencies | N= 1’s and 2’s from Ongoing_  components  _ethics (3.6e) | Merged with 1’s from 4.3e —see derived table |
| 4: Research Partnership Practices | 4.4f Participant recruitment | Ongoing _  RUs_recruit | Nominal; 3 levels | 0= I don’t know  1= Yes  2= No | 0’s and 2’s removed from analysis | Frequencies | N= 1’s and 2’s from Ongoing_  components  _recruit (3.6f) | Merged with 1’s from 4.3f —see derived table |
| 4: Research Partnership Practices | 4.4g Data collection | Ongoing _  RUs_data collection | Nominal; 3 levels | 0= I don’t know  1= Yes  2= No | 0’s and 2’s removed from analysis | Frequencies | N= 1’s and 2’s from Ongoing_  components  _data collection (3.6g) | Merged with 1’s from 4.3g —see derived table |
| 4: Research Partnership Practices | 4.4h Data analysis and interpretation | Ongoing _  RUs_analysis | Nominal; 3 levels | 0= I don’t know  1= Yes  2= No | 0’s and 2’s removed from analysis | Frequencies | N= 1’s and 2’s from Ongoing_  components  _analysis (3.6h) | Merged with 1’s from 4.3h —see derived table |
| 4: Research Partnership Practices | 4.4i Dissemination (i.e., sharing) of research findings to non-academic audiences (e.g., public presentation, summary of findings) | Ongoing _  RUs_dissem  _nonacademics | Nominal; 3 levels | 0= I don’t know  1= Yes  2= No | 0’s and 2’s removed from analysis | Frequencies | N= 1’s and 2’s from Ongoing_  components  _dissem  _nonacademics (3.6i) | Merged with 1’s from 4.3i —see derived table |
| 4: Research Partnership Practices | 4.4j Dissemination of research findings to academic audiences (e.g., peer-reviewed journal publication(s)) | Ongoing _  RUs_dissem  _academics | Nominal; 3 levels | 0= I don’t know  1= Yes  2= No | 0’s and 2’s removed from analysis | Frequencies | N= 1’s and 2’s from Ongoing_  components  _dissem  _academics (3.6j) | Merged with 1’s from 4.3j —see derived table |
| 4: Research Partnership Practices | 4.5 Is/was the research partnership or involvement of research users informed by a theory, model, and/or framework (e.g. Framework for Collaborative Research, Co-KT Framework, etc.)?? | Theory | Nominal; 3 levels | 0= I don’t know  1= Yes  2= No | 0’s and 2’s removed from analysis | Frequencies | N = Eligible (2.1) | / |
| 4: Research Partnership Practices | 4.5a other specification | Theory_specify | Open-ended | Responses will be coded into the available response options or coded into new categories when appropriate. If the response is unclear or there is a small number (<5) of responses in a given category, the responses will be kept as “other”. | | | N= 1’s from Theory (4.5) | / |
| 4: Research Partnership Practices | 4.6 Which components of the of research user involvement are/were informed by theory, model and/or framework? Check all that apply.  4.6a Support of researchers’ ability to involve research users and share power in project decision making | Theory  _phases_  Theory  _phases_ researchers support | Dichotomous | 1= Selected  2= Not selected | 2’s removed from analysis | Frequencies | N= 1’s from Theory (4.5) | / |
| 4: Research Partnership Practices | 4.6b Support of research users’ ability to be involved and share power in project decision making | Theory  _phases_  RUs support | Dichotomous | 1= Selected  2= Not selected | 2’s removed from analysis | Frequencies | N= 1’s from Theory (4.5) | / |
| 4: Research Partnership Practices | 4.6c Maintenance of relationships between researchers and research users | Theory  _phases_  maintain | Dichotomous | 1= Selected  2= Not selected | 2’s removed from analysis | Frequencies | N= 1’s from Theory (4.5) | / |
| 4: Research Partnership Practices | 4.6d Addressing ethical issues | Theory  _phases_  ethics | Dichotomous | 1= Selected  2= Not selected | 2’s removed from analysis | Frequencies | N= 1’s from Theory (4.5) | / |
| 4: Research Partnership Practices | 4.6e Development of funding application | Theory  _phases_  funding | Dichotomous | 1= Selected  2= Not selected | 2’s removed from analysis | Frequencies | N= 1’s from Theory (4.5) | / |
| 4: Research Partnership Practices | 4.6f Setting research priorities | Theory  _phases_  priorities | Dichotomous | 1= Selected  2= Not selected | 2’s removed from analysis | Frequencies | N= 1’s from Theory (4.5) | / |
| 4: Research Partnership Practices | 4.6g Choosing the research questions | Theory  _phases_  RQs | Dichotomous | 1= Selected  2= Not selected | 2’s removed from analysis | Frequencies | N= 1’s from Theory (4.5) | / |
| 4: Research Partnership Practices | 4.6h Development of study design and methods | Theory  _phases_  methods | Dichotomous | 1= Selected  2= Not selected | 2’s removed from analysis | Frequencies | N= 1’s from Theory (4.5) | / |
| 4: Research Partnership Practices | 4.6i Choosing study outcomes | Theory  _phases_  outcomes | Dichotomous | 1= Selected  2= Not selected | 2’s removed from analysis | Frequencies | N= 1’s from Theory (4.5) | / |
| 4: Research Partnership Practices | 4.6j Participant recruitment | Theory  _phases_  recruit | Dichotomous | 1= Selected  2= Not selected | 2’s removed from analysis | Frequencies | N= 1’s from Theory (4.5) | / |
| 4: Research Partnership Practices | 4.6k Data collection | Theory  _phases_  collection | Dichotomous | 1= Selected  2= Not selected | 2’s removed from analysis | Frequencies | N= 1’s from Theory (4.5) | / |
| 4: Research Partnership Practices | 4.6l Data analysis and interpretation | Theory  _phases_  analysis | Dichotomous | 1= Selected  2= Not selected | 2’s removed from analysis | Frequencies | N= 1’s from Theory (4.5) | / |
| 4: Research Partnership Practices | 4.6m Dissemination (i.e., sharing) of research findings to non-academic audiences (e.g., public presentation, summary of findings) | Theory  _phases_  Dissem NAA | Dichotomous | 1= Selected  2= Not selected | 2’s removed from analysis | Frequencies | N= 1’s from Theory (4.5) | / |
| 4: Research Partnership Practices | 4.6n Dissemination of research findings to academic audiences (e.g., peer-reviewed journal publication(s)) | Theory  _phases_  Dissem AA | Dichotomous | 1= Selected  2= Not selected | 2’s removed from analysis | Frequencies | N= 1’s from Theory (4.5) | / |
| 4: Research Partnership Practices | 4.6o Evaluation of research user involvement | Theory  _phases_  eval | Dichotomous | 1= Selected  2= Not selected | 2’s removed from analysis | Frequencies | N= 1’s from Theory (4.5) | / |
| 4: Research Partnership Practices | 4.6p Other, please specify | Theory  _phases_  other | Dichotomous | 1= Selected  2= Not selected | 2’s removed from analysis  1’s get coded | Frequencies | N= 1’s from Theory (4.5) | / |
| 4: Research Partnership Practices | 4.6q I don’t know | Theory  _phases_  IDK | Dichotomous | 1= Selected  2= Not selected | 2’s removed from analysis | Frequencies | N= 1’s from Theory (4.5) | / |
| 4: Research Partnership Practices | 4.6 Other specification | Theory  _phases_  Other specification | Open ended | Responses will be coded into the available response options or coded into new categories when appropriate. If the response is unclear or there is a small number (<5) of responses in a given category, the responses will be kept as “other”. | | | N= 1’s from Theory (4.5) | Merge with 4.6 response options if possible |
| 4: Research Partnership Practices | 4.7 Which approaches or activities are /were used for involving research users in this project? Check all that apply.  4.7a Formal in-person research project meeting(s)- individual and/or group | RU Activities  _  RU Activities  _FiPM | Dichotomous | 1= Selected  2= Not selected | 2’s removed from analysis | Frequencies | N= Eligible (2.1) | / |
| 4: Research Partnership Practices | 4.7b Formal online research project meeting(s)- individual and/or group | RU Activities  _FOM | Dichotomous | 1= Selected  2= Not selected | 2’s removed from analysis | Frequencies | N= Eligible (2.1) | / |
| 4: Research Partnership Practices | 4.7c Formal updates or newsletters about the research project- electronic and/or hard copy | RU Activities  _Fupdates | Dichotomous | 1= Selected  2= Not selected | 2’s removed from analysis | Frequencies | N= Eligible (2.1) | / |
| 4: Research Partnership Practices | 4.7d Informal conversations – in-person, telephone, and/or electronic | RU Activities  _IEC | Dichotomous | 1= Selected  2= Not selected | 2’s removed from analysis | Frequencies | N= Eligible (2.1) | / |
| 4: Research Partnership Practices | 4.7e Distribution of study documents (e.g., protocols, data collection tools) | RU Activities  _distribution | Dichotomous | 1= Selected  2= Not selected | 2’s removed from analysis | Frequencies | N= Eligible (2.1) | / |
| 4: Research Partnership Practices | 4.7f Shared electronic space for saving research project documents (e.g., web portal, password protected site) | RU Activities  _shared Espace | Dichotomous | 1= Selected  2= Not selected | 2’s removed from analysis | Frequencies | N= Eligible (2.1) | / |
| 4: Research Partnership Practices | 4.7g Development of formal documentation or process (e.g., terms of reference, partnership agreements) | RU Activities  _Fdocumentation | Dichotomous | 1= Selected  2= Not selected | 2’s removed from analysis | Frequencies | N= Eligible (2.1) | / |
| 4: Research Partnership Practices | 4.7h Establishment of formal working groups | RU Activities  _Fworking groups | Dichotomous | 1= Selected  2= Not selected | 2’s removed from analysis | Frequencies | N= Eligible (2.1) | / |
| 4: Research Partnership Practices | 4.7i Provision of social opportunities (e.g., research team dinner) | RU Activities  _social opps | Dichotomous | 1= Selected  2= Not selected | 2’s removed from analysis | Frequencies | N= Eligible (2.1) | / |
| 4: Research Partnership Practices | 4.7j Provision of training opportunities and/ or resource materials | RU Activities  _training opps | Dichotomous | 1= Selected  2= Not selected | 2’s removed from analysis | Frequencies | N= Eligible (2.1) | / |
| 4: Research Partnership Practices | 4.7k Honorarium for research users | RU Activities  _honorarium | Dichotomous | 1= Selected  2= Not selected | 2’s removed from analysis | Frequencies | N= Eligible (2.1) | / |
| 4: Research Partnership Practices | 4.7l Reimbursement of expenses for research users | RU Activities  _reimburse | Dichotomous | 1= Selected  2= Not selected | 2’s removed from analysis | Frequencies | N= Eligible (2.1) | / |
| 4: Research Partnership Practices | 4.7m Researchers attending research user meetings or events (e.g. invited talks, fundraising events) | RU Activities  _RU events | Dichotomous | 1= Selected  2= Not selected | 2’s removed from analysis | Frequencies | N= Eligible (2.1) | / |
| 4: Research Partnership Practices | 4.7n Other, please specify | RU Activities  _other | Dichotomous | 1= Selected  2= Not selected | 2’s removed from analysis  1’s get coded | Frequencies | N= Eligible (2.1) | / |
| 4: Research Partnership Practices | 4.7o I don’t know | RU Activities  _IDK | Dichotomous | 1= Selected  2= Not selected | 2’s removed from analysis | Frequencies | N= Eligible (2.1) | / |
| 4: Research Partnership Practices | 4.7p Other specification | RU Activities  _Other specification | Open ended | Responses will be coded into the available response options or coded into new categories when appropriate. If the response is unclear or there is a small number (<5) of responses in a given category, the responses will be kept as “other”. | | | N=1’s from RU_  Activities_Other (4.7o) | Merged with 4.7 responses (if possible) |
| 4: Research Partnership Practices | 4.8 On average, how often are/were research users involved or included in an activity in this project? | Engagement_frequency | Ordinal; 5 levels | 0= I don’t know  1= 1-4 times a year  2= 5-8 times a year  3= 9-12 times a year  4= Other | 0’s removed from analysis  4’s get coded | Frequencies | N= Eligible (2.1) | / |
| 4: Research Partnership Practices | 4.8 Other specification | Other_specify | Open-ended | Responses will be coded into the available response options or coded into new categories when appropriate. If the response is unclear or there is a small number (<5) of responses in a given category, the responses will be kept as “other”. | | | N= 4’s from Engagement_ frequency (4.8) | Merged with 4.8 responses (if possible) |
| 4: Research Partnership Practices | 4.9 Was/will the partnership approach of this project (be) evaluated? | Eval | Nominal; 3 levels | 0= I don’t know  1= Yes  2= No | 0’s removed from analysis | Frequencies | N= Eligible (2.1) | / |
| 4: Research Partnership Practices | 4.10 At what stage of this project was/will the research partnership (be) evaluated? Check all that apply.  4.10a Pre-study | Eval_stage  _  Eval_stage  _Pre | Dichotomous | 1= Selected  2= Not selected | 2’s removed from analysis | Frequencies | N= 1’s from Eval (4.9) | / |
| 4: Research Partnership Practices | 4.10b During the study | Eval_stage  _during | Dichotomous | 1= Selected  2= Not selected | 2’s removed from analysis | Frequencies | N= 1’s from Eval (4.9) | / |
| 4: Research Partnership Practices | 4.10c Post-study | Eval_stage  _post | Dichotomous | 1= Selected  2= Not selected | 2’s removed from analysis | Frequencies | N= 1’s from Eval (4.9) | / |
|  | 4.10d I don’t know | Eval_stage  _IDK | Dichotomous | 1= Selected  2= Not selected | 2’s removed from analysis | Frequencies | N= 1’s from Eval (4.9) | / |
| 4: Research Partnership Practices | 4.10e Other, please specify | Eval_stage  _other | Dichotomous | 1= Selected  2= Not selected | 2’s removed from analysis  1’s get coded | Frequencies | N= 1’s from Eval (4.9) | / |
| 4: Research Partnership Practices | 4.10e Other specification | Eval_stage  _other specification | Open ended | Responses will be coded into the available response options or coded into new categories when appropriate. If the response is unclear or there is a small number (<5) of responses in a given category, the responses will be kept as “other”. | | | N= 1’s from Eval_stage_ other (4.10e) | Merge with 4.10 responses (if possible) |
| 4: Research Partnership Practices | 4.11 What type of partnership evaluation of was/will be conducted? Check all that apply.  4.11a Informal evaluation (for internal use only) | Eval_type  Eval_type_informal | Dichotomous | 1= Selected  2= Not selected | 2’s removed from analysis | Frequencies | N= 1’s from Eval (4.9) | / |
| 4: Research Partnership Practices | 4.11b Formal research study | Eval_type_formal | Dichotomous | 1= Selected  2= Not selected | 2’s removed from analysis | Frequencies | N= 1’s from Eval (4.9) | / |
| 4: Research Partnership Practices | 4.11c I don’t know | Eval_type_IDK | Dichotomous | 1= Selected  2= Not selected | 2’s removed from analysis | Frequencies | N= 1’s from Eval (4.9) | / |
| 4: Research Partnership Practices | 4.11d Other, please specify | Eval_type_other | Dichotomous | 1= Selected  2= Not selected | 2’s removed from analysis  1’s get coded | Frequencies | N= 1’s from Eval (4.9) | / |
| 4: Research Partnership Practices | 4.11d Other specification | Eval_type_other specification | Open ended | Responses will be coded into the available response options or coded into new categories when appropriate. If the response is unclear or there is a small number (<5) of responses in a given category, the responses will be kept as “other”. | | | N= 1’s from Eval (4.9) | / |
| 4: Research Partnership Practices | 4.12 What methodologies were/will be used to evaluate the research partnership in this project? Check all that apply.  4.12a Qualitative | Eval_methods  _  Eval_methods  _Qual | Dichotomous | 1= Selected  2= Not selected | 2’s removed from analysis | Frequencies | N= 1’s from Eval (4.9) | / |
| 4: Research Partnership Practices | 4.12b Quantitative | Eval_methods  _Quant | Dichotomous | 1= Selected  2= Not selected | 2’s removed from analysis | Frequencies | N= 1’s from Eval (4.9) | Merge 1’s with 1’s of 4.12c |
| 4: Research Partnership Practices | 4.12c Mixed methods | Eval_methods  _mixed | Dichotomous | 1= Selected  2= Not selected | 2’s removed from analysis | Frequencies | N= 1’s from Eval (4.9) | Merge 1’s with 1’s of 4.12b |
| 4: Research Partnership Practices | 4.12d Other, please specify | Eval_methods  _other | Dichotomous | 1= Selected  2= Not selected | 2’s removed from analysis  1’s get coded | Frequencies | N= 1’s from Eval (4.9) | / |
| 4: Research Partnership Practices | 4.12e I don’t know | Eval_methods  _IDK | Dichotomous | 1= Selected  2= Not selected | 2’s removed from analysis | Frequencies | N= 1’s from Eval (4.9) |  |
| 4: Research Partnership Practices | 4.12d Other specification | Eval_methods  _Other specification | Open- ended | Responses will be coded into the available response options or coded into new categories when appropriate. If the response is unclear or there is a small number (<5) of responses in a given category, the responses will be kept as “other”. | | | N= 1’s from Eval_methods  _other (4.12d) | Merged with 4.12 responses (if possible |
| 4: Research Partnership Practices | 4.13 Was/will any validated instrument (be) used for the evaluation of the research partnership of this project? | Eval_validated instrument | Nominal; 3 levels | 0= I don’t know  1=Yes  2= No | 0’s removed from analysis | Frequencies | N= 1’s from 4.12b and 4.12c | / |
| 4: Research Partnership Practices | 4.13a Please specify the validated instrument that was/will be used for the evaluation of the research partnership of this project: | Validated instrument_ specify | Open ended | Responses will be coded into the available response options or coded into new categories when appropriate. If the response is unclear or there is a small number (<5) of responses in a given category, the responses will be kept as “other”. | | | N= 1’s from Eval_ validated instrument (4.13) | / |
| 4: Research Partnership Practices | 4.14 Are/were diversity considerations explicitly incorporated when building the partnership? | Considerations | Nominal; 3 levels | 0= I don’t know  1= Yes  2= No | 0’s removed from analysis | Frequencies | N= Eligible (2.1) | / |
| 4: Research Partnership Practices | 4.15 What type(s) of diversity considerations are/were taken into account? Check all that apply.  4.15a Race | Considerations  _  Considerations  _Race | Dichotomous | 1= Selected  2= Not selected | 2’s removed from analysis | Frequencies | N= 1’s from Considerations (4.14) | / |
| 4: Research Partnership Practices | 4.15b Ethnicity | Considerations  _Ethnicity | Dichotomous | 1= Selected  2= Not selected | 2’s removed from analysis | Frequencies | N= 1’s from Considerations (4.14) | / |
| 4: Research Partnership Practices | 4.15c Indigeneity | Considerations  _Indigeneity | Dichotomous | 1= Selected  2= Not selected | 2’s removed from analysis | Frequencies | N= 1’s from Considerations (4.14) | / |
| 4: Research Partnership Practices | 4.15d Gender | Considerations  _Gender | Dichotomous | 1= Selected  2= Not selected | 2’s removed from analysis | Frequencies | N= 1’s from Considerations (4.14) | / |
| 4: Research Partnership Practices | 4.15e Class | Considerations  _class | Dichotomous | 1= Selected  2= Not selected | 2’s removed from analysis | Frequencies | N= 1’s from Considerations (4.14) | / |
| 4: Research Partnership Practices | 4.15f Sexuality | Considerations  _sexuality | Dichotomous | 1= Selected  2= Not selected | 2’s removed from analysis | Frequencies | N= 1’s from Considerations (4.14) | / |
| 4: Research Partnership Practices | 4.15g Geography | Considerations  _geography | Dichotomous | 1= Selected  2= Not selected | 2’s removed from analysis | Frequencies | N= 1’s from Considerations (4.14) | / |
| 4: Research Partnership Practices | 4.15h Age | Considerations  _age | Dichotomous | 1= Selected  2= Not selected | 2’s removed from analysis | Frequencies | N= 1’s from Considerations (4.14) | / |
| 4: Research Partnership Practices | 4.15i Ability | Considerations  _ability | Dichotomous | 1= Selected  2= Not selected | 2’s removed from analysis | Frequencies | N= 1’s from Considerations (4.14) | / |
| 4: Research Partnership Practices | 4.15j Immigration status | Considerations  _immigration | Dichotomous | 1= Selected  2= Not selected | 2’s removed from analysis | Frequencies | N= 1’s from Considerations (4.14) | / |
| 4: Research Partnership Practices | 4.15k Religion | Considerations  _religion | Dichotomous | 1= Selected  2= Not selected | 2’s removed from analysis | Frequencies | N= 1’s from Considerations (4.14) | / |
| 4: Research Partnership Practices | 4.15l Other, please specify | Considerations  _other | Dichotomous | 1= Selected  2= Not selected | 2’s removed from analysis  1’s get coded | Frequencies | N= 1’s from Considerations (4.14) | / |
| 4: Research Partnership Practices | 4.15m I don’t know | Considerations  _IDK | Dichotomous | 1= Selected  2= Not selected | 2’s removed from analysis | Frequencies | N= 1’s from Considerations (4.14) | / |
| 4: Research Partnership Practices | 4.15l Other specification | Considerations  _other specification | Open- ended | Responses will be coded into the available response options or coded into new categories when appropriate. If the response is unclear or there is a small number (<5) of responses in a given category, the responses will be kept as “other”. | | | N= 1’s from considerations  _other (4.15l) | Merged with 4.15 responses (if possible) |
| 4: Research Partnership Practices | 4.16 Is there anything else you would like to tell us about the features and practices of the research partnership and involvement of research users in this project? | Partnership practices_other | Open ended | Responses will be coded into categories when appropriate. | | | N= Eligible (2.1) | / |
| 5: Perceived Effects of Engagement on the Research Process and Outcomes | 5.1 In your opinion, how much did involvement of research users in this project influence the overall project? | Outcome_summary_ completed | Ordinal; 4 levels | 1= No influence  2= A little influence  3= Moderate influence  4= Significant influence | / | Calculate medians and interquartile ranges for summary estimates | N= 2’s from Completed_  Phases (3.4) | Merge responses with 5.2 (see derived table) |
| 5: Perceived Effects of Engagement on the Research Process and Outcomes | 5.2 In your opinion, how much has involvement of research users in this project influenced the overall project to date? | Outcome_summary_ongoing | Ordinal; 4 levels | 1= No influence  2= A little influence  3= Moderate influence  4= Significant influence | / | Calculate medians and interquartile ranges for summary estimates | N= 1’s from Completed_  Phases (3.4) | / |
| 5: Perceived Effects of Engagement on the Research Process and Outcomes | O5.3 Setting the project’s research priorities | Outcome_priorities_ongoing | Ordinal; 4 levels | 1= No influence  2= A little influence  3= Moderate influence  4= Significant influence | / | Frequencies | N= 1’s from Ongoing_RUs  _priorities (4.4a) | Merge responses with C5.3 (see derived table) |
| 5: Perceived Effects of Engagement on the Research Process and Outcomes | O5.4 Choosing the research questions | Outcome_research questions_ongoing | Ordinal; 4 levels | 1= No influence  2= A little influence  3= Moderate influence  4= Significant influence | / | Frequencies | N= 1’s from Ongoing_RUs_  Questions (4.4b) | Merge responses with C5.4 (see derived table) |
| 5: Perceived Effects of Engagement on the Research Process and Outcomes | O5.5 Development of study design and methods | Outcome_methodology_ongoing | Ordinal; 4 levels | 1= No influence  2= A little influence  3= Moderate influence  4= Significant influence | / | Frequencies | N= 1’s from Ongoing_RUs_  Methods (4.4c) | Merge responses with C5.5 (see derived table) |
| 5: Perceived Effects of Engagement on the Research Process and Outcomes | O5.6 Choosing study outcomes | Outcome_study outcomes_ongoing | Ordinal; 4 levels | 1= No influence  2= A little influence  3= Moderate influence  4= Significant influence | / | Frequencies | N= 1’s from Ongoing_RUs_  Questions (4.4d) | Merge responses with C5.6 (see derived table) |
| 5: Perceived Effects of Engagement on the Research Process and Outcomes | O5.7 Development of research ethics documents (*e.g., consent forms*) | Outcome_ethics_ ongoing | Ordinal; 4 levels | 1= No influence  2= A little influence  3= Moderate influence  4= Significant influence | / | Frequencies | N= 1’s from Ongoing_RUs_  Ethics (4.4e) | Merge responses with C5.7 (see derived table) |
| 5: Perceived Effects of Engagement on the Research Process and Outcomes | O5.8 Participant recruitment | Outcome_recruitment_ongoing | Ordinal; 4 levels | 1= No influence  2= A little influence  3= Moderate influence  4= Significant influence | / | Frequencies | N= 1’s from Ongoing_RUs_  Recruit (4.4f) | Merge responses with C5.8 (see derived table) |
| 5: Perceived Effects of Engagement on the Research Process and Outcomes | O5.9 Data collection | Outcome_data collection_ongoing | Ordinal; 4 levels | 1= No influence  2= A little influence  3= Moderate influence  4= Significant influence | / | Frequencies | N= 1’s from Ongoing_RUs_  Data collection (4.4g) | Merge responses with C5.9(see derived table) |
| 5: Perceived Effects of Engagement on the Research Process and Outcomes | O5.10 Data analysis and interpretation | Outcome_analysis_ongoing | Ordinal; 4 levels | 1= No influence  2= A little influence  3= Moderate influence  4= Significant influence | / | Frequencies | N= 1’s from Ongoing_RUs_  Analysis (4.4h) | Merge responses with C5.10 (see derived table) |
| 5: Perceived Effects of Engagement on the Research Process and Outcomes | O5.11 Dissemination (*i.e., sharing*) of research findings to non-academic audiences (*e.g., public presentation, summary of findings*) | Outcome_non-academic dissemination_ ongoing | Ordinal; 4 levels | 1= No influence  2= A little influence  3= Moderate influence  4= Significant influence | / | Frequencies | N= 1’s from Ongoing_RUs_  Dissem_ nonacademics (4.4i) | Merge responses with C5.11 (see derived table) |
| 5: Perceived Effects of Engagement on the Research Process and Outcomes | O5.12 Dissemination of research findings to academic audiences (*e.g., peer-reviewed journal publication(s)*) | Outcome_academic dissemination_ongoing | Ordinal; 4 levels | 1= No influence  2= A little influence  3= Moderate influence  4= Significant influence | / | Frequencies | N= 1’s from Ongoing_RUs_  Dissem_academics (4.4h) | Merge responses with C5.12 (see derived table) |
| 5: Perceived Effects of Engagement on the Research Process and Outcomes | C5.3 Setting the research priorities | Outcome_priorities_completed | Ordinal; 4 levels | 1= No influence  2= A little influence  3= Moderate influence  4= Significant influence | / | Frequencies | N= 1’s from Completed_RUs_ priorities (4.3a) | Merge responses with O5.3 (see derived table) |
| 5: Perceived Effects of Engagement on the Research Process and Outcomes | C5.4 Choosing the research questions | Outcome_research questions_completed | Ordinal; 4 levels | 1= No influence  2= A little influence  3= Moderate influence  4= Significant influence | / | Frequencies | N= 1’s from Completed_RUs_ questions (4.3b) | Merge responses with O5.4 (see derived table) |
| 5: Perceived Effects of Engagement on the Research Process and Outcomes | C5.5 Development of study design and methods | Outcome_methodology_completed | Ordinal; 4 levels | 1= No influence  2= A little influence  3= Moderate influence  4= Significant influence | / | Frequencies | N= 1’s from Completed_RUs_ methods (4.3d) | Merge responses with O5.5 (see derived table) |
| 5: Perceived Effects of Engagement on the Research Process and Outcomes | C5.6 Choosing study outcomes | Outcome_research outcomes_completed | Ordinal; 4 levels | 1= No influence  2= A little influence  3= Moderate influence  4= Significant influence | / | Frequencies | N= 1’s from Completed_RUs_ questions (4.3c) | Merge responses with O5.6 (see derived table) |
| 5: Perceived Effects of Engagement on the Research Process and Outcomes | C5.7 Development of research ethics documents (*e.g., consent forms*) | Outcome_ethics_ completed | Ordinal; 4 levels | 1= No influence  2= A little influence  3= Moderate influence  4= Significant influence | / | Frequencies | N= 1’s from Completed_RUs_ ethics (4.3e) | Merge responses with O5.7 (see derived table) |
| 5: Perceived Effects of Engagement on the Research Process and Outcomes | C5.8 Participant recruitment | Outcome_recruitment_completed | Ordinal; 4 levels | 1= No influence  2= A little influence  3= Moderate influence  4= Significant influence | / | Frequencies | N= 1’s from Completed_RUs_ recruit (4.3f) | Merge responses with O5.8 (see derived table) |
| 5: Perceived Effects of Engagement on the Research Process and Outcomes | C5.9 Data collection | Outcome_data collection_completed | Ordinal; 4 levels | 1= No influence  2= A little influence  3= Moderate influence  4= Significant influence | / | Frequencies | N= 1’s from Completed_RUs_ data collection (4.3g) | Merge responses with O5.9 (see derived table) |
| 5: Perceived Effects of Engagement on the Research Process and Outcomes | C5.10 Data analysis and interpretation | Outcome_analysis_completed | Ordinal; 4 levels | 1= No influence  2= A little influence  3= Moderate influence  4= Significant influence | / | Frequencies | N= 1’s from Completed_RUs_ analysis (4.3h) | Merge responses with O5.10 (see derived table) |
| 5: Perceived Effects of Engagement on the Research Process and Outcomes | C5.11 Dissemination (*i.e., sharing*) of research findings to non-academic audiences (*e.g., public presentation, summary of findings*) | Outcome_non-academic dissemination_completed | Ordinal; 4 levels | 1= No influence  2= A little influence  3= Moderate influence  4= Significant influence | / | Frequencies | N= 1’s from Completed_RUs_ dissem_ nonacademics (4.3i) | Merge responses with O5.11 (see derived table) |
| 5: Perceived Effects of Engagement on the Research Process and Outcomes | C5.12 Dissemination of research findings to academic audiences (*e.g., peer-reviewed journal publication(s)*) | Outcome_academic dissemination_ completed | Ordinal; 4 levels | 1= No influence  2= A little influence  3= Moderate influence  4= Significant influence | / | Frequencies | N= 1’s from Completed_RUs_ dissem_ academics (4.3j) | Merge responses with O5.12(see derived table) |
| 5: Perceived Effects of Engagement on the Research Process and Outcomes | 5.13 In your opinion, to what extent did this project influence research user knowledge and/or skills for engaging in research activities? | Outcome_RU knowledge | Ordinal; 4 levels | 1= No influence  2= A little influence  3= Moderate influence  4= Significant influence | / | Calculate medians and interquartile ranges for summary estimates | N= 2’s from Completed_  Phases (3.4) |  |
| 5: Perceived Effects of Engagement on the Research Process and Outcomes | 5.14 In your opinion, to what extent did this project influence researcher knowledge and/or skills for involving research users in research partnerships? | Outcome_Researcher capacity | Ordinal; 4 levels | 1= No influence  2= A little influence  3= Moderate influence  4= Significant influence | / | Calculate medians and interquartile ranges for summary estimates | N= 2’s from Completed_  Phases (3.4) | / |
| 5: Perceived Effects of Engagement on the Research Process and Outcomes | 5.15 In your opinion, to what extent did this project produce useful research findings for the research users on the team? | Outcome_research user_findings | Ordinal; 4 levels | 1= No influence  2= A little influence  3= Moderate influence  4= Significant influence | / | Calculate medians and interquartile ranges for summary estimates | N= 2’s from Completed_  Phases (3.4) | / |
| 5: Perceived Effects of Engagement on the Research Process and Outcomes | 5.16 In your opinion, to what extent did the project findings influence a specific decision by research users on the team? | Outcome_research user_decisions | Ordinal; 4 levels | 1= No influence  2= A little influence  3= Moderate influence  4= Significant influence | / | Calculate medians and interquartile ranges for summary estimates | N= 2’s from Completed_  Phases (3.4) | / |
| 5: Perceived Effects of Engagement on the Research Process and Outcomes | 5.17 In your opinion, to what extent did the project findings influence a specific action by research users on the team? | Outcome_ research user_practices | Ordinal; 4 levels | 1= No influence  2= A little influence  3= Moderate influence  4= Significant influence | / | Calculate medians and interquartile ranges for summary estimates | N= 2’s from Completed_  Phases (3.4) | / |
| 5: Perceived Effects of Engagement on the Research Process and Outcomes | 5.18 In your opinion, to what extent did this project influence evidence-informed decision making by research users on the team? | Outcome_research-user_policies | Ordinal; 4 levels | 1= No influence  2= A little influence  3= Moderate influence  4= Significant influence | / | Calculate medians and interquartile ranges for summary estimates | N= 2’s from Completed_  Phases (3.4) | / |
| 5: Perceived Effects of Engagement on the Research Process and Outcomes | In your opinion, to what extent do you believe how much has/did involvement of research users in this project influence(d) the following:  5.19 The production of more useful research findings in the field | Outcome-overall  Outcome-overall-findings | Ordinal; 4 levels | 1= No influence  2= A little influence  3= Moderate influence  4= Significant influence | / | Calculate medians and interquartile ranges for summary estimates | N= 2’s from Completed_  Phases (3.4) | / |
| 5: Perceived Effects of Engagement on the Research Process and Outcomes | 5.20 The promotion of evidence-informed decision making in health care or the health system | Outcome-overall-decisions | Ordinal; 4 levels | 1= No influence  2= A little influence  3= Moderate influence  4= Significant influence | / | Calculate medians and interquartile ranges for summary estimates | N= 2’s from Completed_  Phases (3.4) | / |
| 5: Perceived Effects of Engagement on the Research Process and Outcomes | 5.21 The projects’ impact on health care professional practices | Outcome_overall_practices | Ordinal; 4 levels | 1= No influence  2= A little influence  3= Moderate influence  4= Significant influence | / | Calculate medians and interquartile ranges for summary estimates | N= 2’s from Completed_  Phases (3.4) | / |
| 5: Perceived Effects of Engagement on the Research Process and Outcomes | 5.22 The projects’ impact on health system policies | Outcome_overall_policies | Ordinal; 4 levels | 1= No influence  2= A little influence  3= Moderate influence  4= Significant influence | / | Calculate medians and interquartile ranges for summary estimates | N= 2’s from Completed_  Phases (3.4) | / |
| 5: Perceived Effects of Engagement on the Research Process and Outcomes | 5.23 Other, please specify: | Outcome_others | Open ended | Responses will be coded into the available response options or coded into new categories when appropriate. If the response is unclear or there is a small number (<5) of responses in a given category, the responses will be kept as “other”. | | | N= 2’s from Completed_  Phases (3.4) | Merge if possible |
| 5: Perceived Effects of Engagement on the Research Process and Outcomes | 5.24 Is there anything else you would like to tell us about your views on the effects of involving research users in this project? | Outcome-other | Open ended | Responses will be coded into categories when appropriate. | | | N= Eligible (2.1) | / |
| 6: Teamwork in the Partnership | 6.1 Most members of this research team fit what I believe to be the ideal team member | Co_ideal team member | Ordinal; 5 levels | 1= Strongly disagree  2=Disagree  3=Neutral  4= Agree  5= Strongly agree | / | / | N= Eligible (2.1) | Merge all cohesion questions (6.1-6.9) (See derived table) |
| 6: Teamwork in the Partnership | 6.2 I feel that I am/was sufficiently included by the research team in all the team’s activities | Co_inclusion | Ordinal; 5 levels | 1= Strongly disagree  2=Disagree  3=Neutral  4= Agree  5= Strongly agree | / | / | N= Eligible (2.1) | Merge all cohesion questions (6.1-6.9) (See derived table) |
| 6: Teamwork in the Partnership | 6.3 I find/found most of the activities in which I participate/ participated as a member of this team rewarding | co_rewarding activities | Ordinal; 5 levels | 1= Strongly disagree  2=Disagree  3=Neutral  4= Agree  5= Strongly agree | / | / | N= Eligible (2.1) | Merge all cohesion questions (6.1-6.9) (See derived table) |
| 6: Teamwork in the Partnership | 6.4 If some members of the research team decided to dissolve the team by leaving, I would (have) try/tried to dissuade them | co_team dissolve | Ordinal; 5 levels | 1= Strongly disagree  2=Disagree  3=Neutral  4= Agree  5= Strongly agree | / | / | N= Eligible (2.1) | Merge all cohesion questions (6.1-6.9) (See derived table) |
| 6: Teamwork in the Partnership | 6.5 If asked to participate in another project like this one, I would like to be with the same people who are/were involved in this project | co_future work | Ordinal; 5 levels | 1= Strongly disagree  2=Disagree  3=Neutral  4= Agree  5= Strongly agree | / | / | N= Eligible (2.1) | Merge all cohesion questions (6.1-6.9) (See derived table) |
| 6: Teamwork in the Partnership | 6.6 I like/liked the research team I am/was working with | co_liking | Ordinal; 5 levels | 1= Strongly disagree  2=Disagree  3=Neutral  4= Agree  5= Strongly agree | / | / | N= Eligible (2.1) | Merge all cohesion questions (6.1-6.9) (See derived table) |
| 6: Teamwork in the Partnership | 6.7 I think our research team meets/met frequently enough | co_meeting | Ordinal; 5 levels | 1= Strongly disagree  2=Disagree  3=Neutral  4= Agree  5= Strongly agree | / | / | N= Eligible (2.1) | Merge all cohesion questions (6.1-6.9) (See derived table) |
| 6: Teamwork in the Partnership | 6.8 I feel that working with this particular research team enables/ enabled me to attain my personal goals for which the team was formed | co_attain goals | Ordinal; 5 levels | 1= Strongly disagree  2=Disagree  3=Neutral  4= Agree  5= Strongly agree | / | / | N= Eligible (2.1) | Merge all cohesion questions (6.1-6.9) (See derived table) |
| 6: Teamwork in the Partnership | 6.9 Compared to other research teams, my team works/ worked well together | co_works well | Ordinal; 5 levels | 1= Strongly disagree  2=Disagree  3=Neutral  4= Agree  5= Strongly agree | / | / | N= Eligible (2.1) | Merge all cohesion questions (6.1-6.9) (See derived table) |
| 6: Teamwork in the Partnership | 6.10 Is there anything else you would like to tell us about your views on how researchers and research users worked together as a team for this project? | co_other | Open ended | Responses will be coded into categories when appropriate. | | | N= Eligible (2.1) | / |
| 7: Working in Research Partnerships | 7.1 I have the knowledge and skills to engage in research partnerships | Cap_summary | Ordinal; 5 levels | 1= Strongly disagree  2=Disagree  3=Neutral  4= Agree  5= Strongly agree | / | Calculate mean scores for Capacity domain. The scores would indicate the average agreement of participants for each domain | N1= 1’s (researchers) from Role (2.2)  N2= 2’s (RUs) from Role (2.2) | / |
| 7: Working in Research Partnerships | 7.2 I am confident in my ability to engage in research partnerships | Motivation_ability | Ordinal; 5 levels | 1= Strongly disagree  2=Disagree  3=Neutral  4= Agree  5= Strongly agree | / | / | N1= 1’s (researchers) from Role (2.2)  N2= 2’s (RUs) from Role (2.2) | Merge Motivation domain (7.2; 7.5; 7.6; 7.7; 7.9; 7.10) See derived table |
| 7: Working in Research Partnerships | 7.3 I have the resources (*e.g., time and money)* to engage in research partnerships | Opp_resources | Ordinal; 5 levels | 1= Strongly disagree  2=Disagree  3=Neutral  4= Agree  5= Strongly agree | / | / | N1= 1’s (researchers) from Role (2.2)  N2= 2’s (RUs) from Role (2.2) | Merge Opportunity domain (7.3; 7.4; 7.8) See derived table) |
| 7: Working in Research Partnerships | 7.4 I have support from others (*e.g., colleagues, friends, own organization, etc.)* to engage in research partnerships | Opp_support | Ordinal; 5 levels | 1= Strongly disagree  2=Disagree  3=Neutral  4= Agree  5= Strongly agree | / | / | N1= 1’s (researchers) from Role (2.2)  N2= 2’s (RUs) from Role (2.2) | Merge Opportunity domain (7.3; 7.4; 7.8) See derived table) |
| 7: Working in Research Partnerships | 7.5 There is value in engaging in research partnerships | Motivation_value | Ordinal; 5 levels | 1= Strongly disagree  2=Disagree  3=Neutral  4= Agree  5= Strongly agree | / | / | N1= 1’s (researchers) from Role (2.2)  N2= 2’s (RUs) from Role (2.2) | Merge Motivation domain (7.2; 7.5; 7.6; 7.7; 7.9; 7.10) See derived table |
| 7: Working in Research Partnerships | 7.6 It is my responsibility to engage in research partnerships | Motivation_responsibility | Ordinal; 5 levels | 1= Strongly disagree  2=Disagree  3=Neutral  4= Agree  5= Strongly agree | / | / | N1= 1’s (researchers) from Role (2.2)  N2= 2’s (RUs) from Role (2.2) | Merge Motivation domain (7.2; 7.5; 7.6; 7.7; 7.9; 7.10) See derived table |
| 7: Working in Research Partnerships | 7.7 I intend to engage in research partnerships in future | Motivation_intent | Ordinal; 5 levels | 1= Strongly disagree  2=Disagree  3=Neutral  4= Agree  5= Strongly agree | / | / | N1= 1’s (researchers) from Role (2.2)  N2= 2’s (RUs) from Role (2.2) | Merge Motivation domain (7.2; 7.5; 7.6; 7.7; 7.9; 7.10) See derived table |
| 7: Working in Research Partnerships | 7.8 I feel pressure to engage in research partnerships | Opp_pressure | Ordinal; 5 levels | 1= Strongly disagree  2=Disagree  3=Neutral  4= Agree  5= Strongly agree | / | / | N1= 1’s (researchers) from Role (2.2)  N2= 2’s (RUs) from Role (2.2) | Merge Opportunity domain (7.3; 7.4; 7.8) See derived table) |
| 7: Working in Research Partnerships | 7.9 The decision to engage in research partnerships is beyond my control | Motivation_control | Ordinal; 5 levels | 1= Strongly disagree  2=Disagree  3=Neutral  4= Agree  5= Strongly agree | / | / | N1= 1’s (researchers) from Role (2.2)  N2= 2’s (RUs) from Role (2.2) | Merge Motivation domain (7.2; 7.5; 7.6; 7.7; 7.9; 7.10) See derived table |
| 7: Working in Research Partnerships | 7.10 It is useful to engage in research partnerships | Motivation_useful | Ordinal; 5 levels | 1= Strongly disagree  2=Disagree  3=Neutral  4= Agree  5= Strongly agree | / | / | N1= 1’s (researchers) from Role (2.2)  N2= 2’s (RUs) from Role (2.2) | Merge Motivation domain (7.2; 7.5; 7.6; 7.7; 7.9; 7.10) See derived table |
| 7: Working in Research Partnerships | 7.11 Is there anything else you would like to tell us about your views on your experience working in research partnerships? | Motivation_other | Open ended | Responses will be coded into categories when appropriate. | | | N1= 1’s (researchers) from Role (2.2)  N2= 2’s (RUs) from Role (2.2) | / |
| 8: Research User Experiences | 8.1 I feel/felt comfortable contributing my knowledge, experiences, preferences, values or opinions in this project | RU_comfort | Ordinal; 5 levels | 1= Strongly disagree  2=Disagree  3=Neutral  4= Agree  5= Strongly agree | / | / | N= 2’s (RUs) from Role (2.2) | Merge respect domain question (8.1; 8.2) See derived table |

Appendix 3. Perceived influence of research user involvement in completed research

| Outcome | Perceived influence of research user involvement [n (%)] | | | | |
| --- | --- | --- | --- | --- | --- |
|  | None | A little | Moderate | Significant | *Missing* |
| Overall influence (n=589) | 15 (3%) | 59 (10%) | 161 (27%) | 313 (53%) | *41 (7%)* |
| Setting research project's priorities (n=458) | 6 (1%) | 61 (13%) | 153 (33%) | 208 (45%) | *30 (7%)* |
| Choosing the research questions (n= 412) | 8 (2%) | 75 (18%) | 133 (32%) | 170 (41%) | *26 (6%)* |
| Developing study design and methods (n=377) | 20 (5%) | 89 (24%) | 122 (32%) | 118 (31%) | *28 (7%)* |
| Choosing study outcomes (n=386) | 13 (3%) | 63 (16%) | 144 (37%) | 140 (36%) | *26 (7%)* |
| Developing research ethics documents (n= 195) | 16 (8%) | 41 (21%) | 59 (30%) | 63 (32%) | *16 (8%)* |
| Participant recruitment (n=315) | 7 (2%) | 19 (6%) | 81 (26%) | 183 (58%) | *20 (8%)* |
| Data collection (n=268) | 13 (5%) | 43 (16%) | 62 (23%) | 123 (46%) | *27 (10%)* |
| Data analysis and interpretation (n=314) | 17 (5%) | 64 (20%) | 106 (34%) | 103 (33%) | *24 (8%)* |
| Disseminating findings to non-academic audiences (n=367) | 11 (3%) | 43 (12%) | 117 (32%) | 172 (47%) | *20 (7%)* |
| Disseminating findings to academic audiences (n=292) | 11 (4%) | 79 (27%) | 95 (33%) | 90 (31%) | *17 (6%)* |
| The production of useful research findings in the field (n=290) | 10 (3%) | 41 (14%) | 91 (31%) | 125 (43 %) | *23 (8%)* |
| The promotion of evidence-informed decision making in health care or the health system (n= 290) | 31 (11%) | 66 (23%) | 89 (31%) | 81 (28%) | *23 (8%)* |
| The project's impact on health care professional practices (n=290) | 45 (16%) | 78 (27%) | 80 (28%) | 61 (21%) | *26 (9%)* |
| The project's impact on health system policies (n=290) | 61 (24%) | 94 (32%) | 78 (27%) | 33 (11%) | *24 (8%)* |

Appendix 4. Perceived team cohesion by gender

| Variable | Level of agreement [n (%)] | | | Chi-square, p value |
| --- | --- | --- | --- | --- |
|  | Full sample (n=498) | Gender | |  |
|  |  | Woman (n=328) | Man (n=170) |  |
| Most fit vision of ideal team member (n=490)       Agree       Neutral       Disagree | 399 (81.4)  67 (13.7)  24 (4.9) | 269 (83.5)  37 (11.5)  16 (5.0) | 130 (77.4)  30 (17.9)  8 (4.8) | X^2^=3.80, p=0.15 |
| I felt sufficiently included in all activities (n=492)  Agree       Neutral       Disagree | 439 (89.2)  37 (7.5)  16 (3.3) | 290 (89.8)  25 (7.7)  8 (2.5) | 149 (88.2)  12 (7.1)  8 (4.7) | X^2^=1.83, p=0.40 |
| Most activities were rewarding (n=491)       Agree       Neutral       Disagree | 429 (87.4)  46 (9.4)  16 (3.3) | 281 (87.3)  30 (9.3)  11 (3.4) | 148 (87.6)  16 (9.5)  5 (3.0) | X^2^=0.08, p=0.96 |
| If some members of team dissolved I would persuade them (n=484)       Agree       Neutral       Disagree | 284 (58.7)  138 (28.5)  62 (12.8) | 192 (60.4)  86 (27.0)  40 (12.6) | 92 (55.4)  52 (31.3)  22 (13.3) | X^2^=1.20, p=0.55 |
| I would participate again with the same team (n=489)       Agree       Neutral       Disagree | 380 (77.7)  69 (14.1)  40 (8.2) | 250 (77.9)  46 (14.3)  25 (7.8) | 130 (77.4)  23 (13.7)  15 (8.9) | X^2^=0.21, p=0.90 |
| I liked the research team (n=486)       Agree       Neutral       Disagree | 436 (89.6)  39 (8.0)  11 (2.3) | 285 (89.1)  28 (8.8)  7 (2.2) | 151 (91.0)  11 (6.6)  4 (2.4) | X^2^=0.68, p=0.71 |
| I think our team meets frequently enough (n=486)       Agree       Neutral       Disagree | 374 (77.0)  71 (14.6)  41 (8.4) | 248 (77.7)  46 (14.4)  25 (7.8) | 126 (75.4)  25 (15.0)  16 (9.6) | X^2^=0.49, p=0.78 |
| Working with this team enables my personal goals (n=487)       Agree       Neutral       Disagree | 382 (78.4)  77 (15.8)  28 (5.7) | 247 (77.4)  54 (16.9)  18 (5.6) | 135 (80.4)  23 (13.7)  10 (6.0) | X^2^=0.87, p=0.65 |
| Compared to other teams this team worked well (n=488)       Agree       Neutral       Disagree | 392 (80.3)  70 (14.3)  26 (5.3) | 258 (80.6)  45 (14.1)  17 (5.3) | 134 (79.8)  25 (14.9)  9 (5.4) | X^2^=0.06, p=0.97 |

Appendix 5. Individual behavioral determinants for working in partnership by gender

| Determinant | Level of agreement [n (%)] | | | Chi-square, p value |
| --- | --- | --- | --- | --- |
|  | Full sample (n=498) | Gender | |  |
|  |  | Woman  (n=328) | Man (n=170) |  |
| I have the knowledge and skills to engage (n=495)       Agree       Neutral       Disagree | 476 (96.2)  15 (3.0)  4 (0.8) | 316 (97.2)  7 (2.2)  2 (0.6) | 160 (94.1)  8 (4.7)  2 (1.2) | X^2^=2.95, p=0.23 |
| I am confident in my ability to engage (n=495)  Agree       Neutral       Disagree | 465 (93.9)  25 (5.1)  5 (1.0) | 308 (94.8)  14 (4.3)  3 (0.9) | 157 (92.4)  11 (6.5)  2 (1.2) | X^2^=1.17, p=0.56 |
| I have the resources to engage (n=494)       Agree       Neutral       Disagree | 290 (58.7)  122 (24.7)  82 (16.6) | 176 (54.3)  86 (26.5)  62 (19.1) | 114 (67.1)  36 (21.2)  20 (11.8) | X^2^=8.03, p=0.02 |
| I have the support to engage (n=495)       Agree       Neutral       Disagree | 385 (77.8)  82 (16.6)  28 (5.7) | 249 (76.6)  56 (17.2)  20 (6.2) | 136 (80.0)  26 (15.3)  8 (4.7) | X^2^=0.83, p=0.66 |
| There is value in engaging (n=493)       Agree       Neutral       Disagree | 481 (97.6)  11 (2.2)  1 (0.2) | 317 (98.1)  6 (1.9)  0 (0.0) | 164 (96.5)  5 (2.9)  1 (0.6) | X^2^=2.52, p=0.28 |
| It is my responsibility to engage (n=494)       Agree       Neutral       Disagree | 449 (90.9)  38 (7.7)  7 (1.4) | 302 (92.9)  20 (6.2)  3 (0.9) | 147 (87.0)  18 (10.7)  4 (2.4) | X^2^=4.99, p=0.08 |
| I intend to engage in the future (n=493)       Agree       Neutral       Disagree | 452 (91.7)  32 (6.5)  9 (1.8) | 293 (90.7)  23 (7.1)  7 (2.2) | 159 (93.5)  9 (5.3)  2 (1.2) | X^2^=1.27, p=0.53 |
| I feel pressure to engage (n=495)       Agree       Neutral       Disagree | 140 (28.3)  125 (25.3)  230 (46.5) | 94 (28.9)  75 (23.1)  156 (48.0) | 46 (27.1)  50 (29.4)  74 (43.5) | X^2^=2.39, p=0.30 |
| The decision to engage is beyond my control (n=495)       Agree       Neutral       Disagree | 43 (8.7)  96 (19.4)  356 (71.9) | 29 (8.9)  65 (20.0)  231 (71.1) | 14 (8.2)  31 (18.2)  125 (73.5) | X^2^=0.33, p=0.85 |
| It is useful to engage (n=495)       Agree       Neutral       Disagree | 470 (94.9)  21 (4.2)  4 (0.8) | 311 (95.7)  14 (4.0)  1 (0.3) | 159 (93.5)  8 (4.7)  3 (1.8) | X^2^=3.12, p=0.21 |
